# EB1 Is Required for Spindle Symmetry in Mammalian Mitosis

**DOI:** 10.1371/journal.pone.0028884

**Published:** 2011-12-21

**Authors:** Anke Brüning-Richardson, Kelly J. Langford, Peter Ruane, Tracy Lee, Jon M. Askham, Ewan E. Morrison

**Affiliations:** Leeds Institute of Molecular Medicine, University of Leeds, St. James's University Hospital, Leeds, United Kingdom; Institute of Molecular and Cell Biology, Singapore

## Abstract

Most information about the roles of the adenomatous polyposis coli protein (APC) and its binding partner EB1 in mitotic cells has come from siRNA studies. These suggest functions in chromosomal segregation and spindle positioning whose loss might contribute to tumourigenesis in cancers initiated by APC mutation. However, siRNA-based approaches have drawbacks associated with the time taken to achieve significant expression knockdown and the pleiotropic effects of EB1 and APC gene knockdown. Here we describe the effects of microinjecting APC- or EB1- specific monoclonal antibodies and a dominant-negative EB1 protein fragment into mammalian mitotic cells. The phenotypes observed were consistent with the roles proposed for EB1 and APC in chromosomal segregation in previous work. However, EB1 antibody injection also revealed two novel mitotic phenotypes, anaphase-specific cortical blebbing and asymmetric spindle pole movement. The daughters of microinjected cells displayed inequalities in microtubule content, with the greatest differences seen in the products of mitoses that showed the severest asymmetry in spindle pole movement. Daughters that inherited the least mobile pole contained the fewest microtubules, consistent with a role for EB1 in processes that promote equality of astral microtubule function at both poles in a spindle. We propose that these novel phenotypes represent APC-independent roles for EB1 in spindle pole function and the regulation of cortical contractility in the later stages of mitosis. Our work confirms that EB1 and APC have important mitotic roles, the loss of which could contribute to CIN in colorectal tumour cells.

## Introduction

Since it was first linked with colorectal cancer in 1991 [1], [2] the adenomatous polyposis coli protein (APC) has been extensively investigated. In particular, its role as a tumour suppressor protein whose loss of function leads to the development of colorectal cancer has attracted much attention, eventually identifying a pivotal role for APC in the WNT signalling pathway [3], [4]. However, it is now clear that APC is a multifunctional protein with additional roles to play within cells, for example in cell motility and mitosis [5], [6]. An interaction between APC and one of its binding partners, EB1, is thought to play a crucial part in these processes.

The microtubule (MT) plus end-binding protein EB1 was first identified in a yeast-2-hybrid screen for APC binding partners [7]. It is a highly conserved eukaryotic protein best known for its ability to localise to growing MT plus ends [8], [9] and it therefore belongs to a group of proteins referred to as +TIPS [5], [10]. The interaction between EB1 and APC is modulated by APC phosphorylation [11], [12], though APC can bind directly to MTs independently of EB1, using a region located around a basic domain at the C-terminus of the protein [13]–[16]. The *APC* mutations typically found in colorectal cancers result in the loss of both the MT and the EB1 interaction domains of the protein. In addition to the well-researched APC-EB1 interaction other interactants of EB1 have been discovered in recent years. They can be divided into two major groups according to their shared structural domains, the cytoskeleton-associated protein-glycine-rich (CAP-Gly) domain proteins which include cytoplasmic linker proteins (CLIPs) and the large subunit of the dynactin complex (p150Glued) and the SxIP proteins, which all share a short and conserved motif. Members of this group of proteins include (apart from APC) the CLIP associated proteins (CLASPs), the mitotic centromere associated kinesin (MCAK), TIP150, the microtubule-actin crosslinking factor (MACF), the stromal interaction molecule 1 (STIM1), p140Cap, Navigators, melanophillin, RhoGEF2, CDK5RAP2 and DDA3. [17]–[24].

During mitosis EB1 has also been demonstrated to interact at the centrosome with the FGFR1 oncogene partner (FOP) in combination with CAP350 to form a complex essential for MT anchorage [25].

Much information has been gathered about the roles of APC and EB1 with regard to their interactions with MTs in interphase cells [5], [6]. Recent studies also imply that the binding of APC to EB1 relieves a self-inhibitory EB1 configuration, allowing the localisation of EB1 to MT tips where it promotes plus end growth by inhibiting MT catastrophe [26]. However, the roles of APC and EB1 and the potential functional significance of their interaction in mitotic cells are not fully understood. Nevertheless, loss of these functions may be of importance in the development of colorectal cancer. In particular, the concept that APC mutation contributes to the genetic instability essential for the progression from benign polyp to aggressive carcinoma is compelling [27]–[30]. Aneuploidy in these tumours may be driven by chromosomal instability (CIN), which is common in colorectal cancers initiated by APC mutation [31] and may result from defects in chromosomal segregation during mitosis. In addition, studies in non-mammalian systems such as *Drosophila* and yeast indicate that APC (or putative APC orthologues) and EB1 are involved in the process of spindle positioning [5]. Interestingly, recent publications indicate that EB1 may play a bigger role in cancers than previously believed. Liu et al. [32] concluded that EB1 could act as an oncogene through its involvement in the activation of the WNT signalling pathway in head and neck cancers and Orimo et al. [33] suggested that EB1 might represent a prognostic biomarker in hepatocellular carcinoma. Most recently, Dong et al. [34] proposed an oncogenic role for EB1 in breast cancer. In this study a correlation between EB1 levels and clinicopathological parameters was found. EB1 siRNA in breast cancer cell lines was characterised by inhibition of cell proliferation and EB1 overexpression promoted cell proliferation. Our current understanding of the roles of the two proteins in mammalian cell mitosis is mainly based on recent siRNA studies. APC or EB1 knockdown was found to cause an increased incidence of defects in chromosomal segregation and errors in processes such as spindle alignment [35]–[37], but the mechanistic basis of these observations remains unclear. Furthermore, Rusan and Peifer [38] have noted that there are discrepancies in the findings from different groups in siRNA studies, which complicates the interpretation of their results. In addition to the results from the siRNA studies in mammalian systems various authors have revealed mitotic roles of APC and EB1 from observations in *Xenopus* extracts. Dikovskaya et al. [39] described a role for APC in centrosomally driven spindle formation, Zhang et al. [40] revealed an APC/EB1 interaction in association with BubR1 during metaphase chromosome alignment.

In this study we investigated the roles of APC and EB1 in mitotic mammalian cells using antibody and recombinant protein microinjection. We reasoned that this approach could complement previous siRNA studies by allowing the targeting of specific regions within, or interactions of, these proteins. In addition, this approach would permit rapid visualisation of phenotypic effects in injected mitotic cells without the unavoidable time lags associated with the siRNA methodology or the expression of dominant-negative mutants, where mitotic phenotypes might result indirectly from effects initiated in interphase cells. It would also avoid the problem of identifying controls for the increased levels of ß-catenin that should result from loss of APC function in the WNT signalling pathway, an important consideration given recent observations [41].

This approach has revealed a number of specific and novel phenotypes, including mitotic stage-specific cortical blebbing and asymmetric spindle pole movement following injection with EB1 antibody or dominant-negative EB1 protein. The defects identified in our studies are consistent with important mitotic roles for APC and EB1, the loss of which could contribute to CIN in colorectal tumour cells.

## Results

### Antibody characterisation

The EB1-specific mouse monoclonal antibody 1A11 was prepared in collaboration with the Cancer Research UK (CRUK) Research Monoclonal Antibody Service (RMAS). One of the driving factors behind its production was the observation that most commercially available anti-EB1 mouse monoclonal antibodies recognise epitopes near the *C*-terminus of the protein. Therefore, to generate a reagent that would recognise an epitope outside of this region, the immunogen used to produce 1A11 was the EB1 deletion mutant fusion protein GST-EB1-ΔC1. This protein lacks the final 50aa in EB1 [17]. Antibody 1A11 was identified using purified 6his-EB1 [17] in an ELISA screen of hybridoma supernatants. This antibody is now commercially available (ref.2164, Cell Signaling Technology, Inc) and was shown to specifically react with a 30 kDa protein by Western blot.

**Figure 1:**
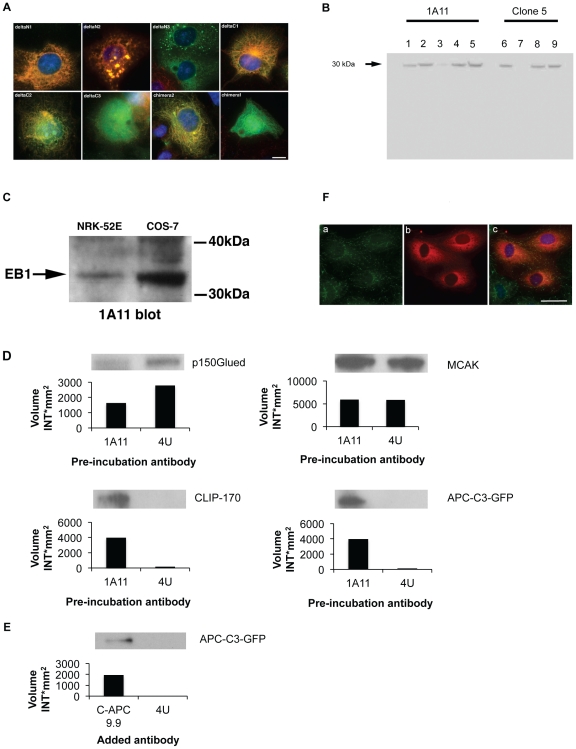
Functional characterisation of antibodies used in this study. Panel A. The epitope recognised by the EB1-specific monoclonal antibody 1A11 was mapped by co-immunostaining of COS-7 cells transiently transfected with a GFP-tagged EB1 deletion series (Table 1, [17]) and EB1/EB2-GFP chimeric proteins using anti-GFP antibodies (green) and the 1A11 antibody (red). DNA was counterstained using DAPI (blue). The presence of the 1A11 epitope was confirmed by co-localisation of GFP and 1A11 staining. Chimera1 is an EB1/EB2 hybrid protein, while chimera 2 is an EB2/EB1 hybrid. This analysis indicated that the 1A11 epitope lies between aa131–168. Scale bar = 10 µm. Panel B. Specificity of antibody 1A11 is revealed by RNAi experiments. Binding of EB1 is greatly reduced in lysates after EB1 knockdown (lane 3), but not after EB2 (lane 4) and EB3 (lane 5) knockdown. Binding to EB1 is also present in the negative control (scrambled RNA, lane 2) and in normal HeLa cell lysates (lane 1). Similar results are obtained with commercial EB1 antibody clone 5. EB1 knockdown (lane 7) reduced binding of this antibody, whereas knockdown of EB2 (lane 8) or EB3 (lane 7) or with scrambled oligos (lane 6) did not affect EB1 binding. Panel C. EB1 is recognized in lysates from COS7 and NRK-52E cells by 1A11 after Western blotting. Panel D. Effects of 1A11 binding on EB1 interactions with ligands. GST-EB1 bound to glutathione-agarose beads was pre-incubated with 1A11 or non-specific IgG (4 U) before being used in pull-downs from cell extracts. Bound proteins in each pull down were revealed by SDS-PAGE and Western blotting and the effects of antibody pre-incubation compared. Protein levels were quantified using Quantity One and are shown in histograms after background subtraction, corresponding band patterns are shown above the histograms. 1A11 pre-incubation inhibited the binding of GST-EB1 to p150Glued, had little detectable effect on the association with MCAK, and promoted the interactions with CLIP-170 and APC-C3-GFP, an APC C-terminal fragment containing the EB1- and MT-binding regions in APC. Panel E. Effects of the C-APC 9.9 antibody on the EB1 interaction with APC. The anti-APC antibody C-APC9.9 or control IgG 4 U was added to extracts prepared from cells expressing APC-C3-GFP. The ability of GST-EB1 to precipitate APC-C1-GFP from these extracts was then examined. The binding of C-APC9.9 to its epitope in the MT-binding region of APC enhanced the interaction between GST-EB1 and APC-C1-GFP. Protein levels were quantified by Quantity One and presented as histograms after background subtraction, corresponding band patterns are shown above the histogram. Panel F. Endogenous EB1 is not displaced from MT ends in NRK-52E cells microinjected with 1A11. Immunofluorescence images reveal localization of EB1 at MT ends after cells microinjected with 1A11 were fixed and stained with the EB1 antibody clone 5. Fluorescently labelled dextran (red; panel b) indicates a successful microinjection. Immunostaining with clone 5 (green; panel a) reveals EB1 at MT ends. DNA was counterstained using DAPI (blue). Scale bar = 10 µM.

In addition to the published data to further define the epitope recognised by 1A11 we performed an immunostaining screen against a panel of GFP-tagged EB1 deletion mutants (see Table 1 in Askham et al. [17]), and chimeric EB1/EB2 proteins expressed in transfected cells (Figure 1A). Co-localisation with 1A11 staining indicated that the epitope fell outside EB1 aa regions 1–113 (deltaC3, no co-localisation) and regions 150–268 (deltaN3), which was further narrowed down by co-localisation studies with the EB1/EB2 chimeras. This indicated that the 1A11 epitope was located in a region encompassed by aa131–168. To investigate the specificity of this antibody we carried out RNAi experiments. HeLA cells were used for EB1, EB2 and EB3 knockdown experiments. Lysates from these were analysed with the EB1 antibody 1A11 and as a control a commercially available EB1 antibody, clone 5 (BD Transduction Laboratories), by Western blotting. Binding of both EB1 antibodies was greatly reduced in the lysates from the EB1 knockdowns, but there was strong binding to EB1 in the lysates from the EB2 and EB3 knockdowns. Strong EB1 binding was also observed in the untreated lysate or the negative control lysate (scrambled) when 1A11 was used (Figure 1B).

**Table 1:** Mitotic success rates following antibody microinjection.

Microinjected antibody	Number ofmicroinjected cells (n)	Mitosis	Rate (%)
4 U	16	Failed	25
		Completed	75
1A11	19	Failed	41.1
		Completed	58.9
ALI 12–28	16	Failed	43.7
		Completed	56.3
C-APC 9.9	18	Failed	11.1
		Completed	88.9
C-APC 28.9	15	Failed	26.7
		Completed	73.3

This table summarises the percentage of microinjected NRK-52E cells that completed mitosis within 2 h of injection. Although the majority of microinjected cells completed mitosis successfully, cells microinjected with 1A11 or ALI 12–28 had a slightly lower success rate than cells injected with the control antibody 4 U or with C-APC 9.9 and C-APC 28.9. However, statistical analysis indicated that this was not a significant effect. The failed category includes cell that had started mitosis but did not finish within 2 h of microinjection and cells that did not progress in mitosis after microinjection.

Western blot analysis of COS7 cells and the cell line used in this study, NRK-52E, with 1A11 and clone 5 (not shown) revealed the presence of endogenous EB1 (Figure 1C, also Figure S1). We next examined whether the binding of 1A11 to EB1 inhibited its interactions with some of its known ligands. The acidic tail in EB1 is known to be important for its interaction with CLIP-170, but is thought to be a less important factor in the interaction between EB1 and p150Glued [42], [43]. In turn, the interaction between EB1 and APC appears to require an EB1 region more *N*-terminal to that used by p150Glued [17], [26], [44]. A screen against these three proteins therefore covers the major binding modes currently identified for EB1 ligands. Finally, we have recently identified an association between EB1 and the mitotic kinesin MCAK [45]. It is known that these major interactions occur during interphase and mitosis, therefore, we wanted to assess the inhibitory effect of 1A11 on EB1 interactions in cell extracts of NRK-E52 cells. We reasoned that any observed effect of 1A11 on binding patterns could have implications for any mitotic phenotypes seen in microinjected cells. To assess the effect of 1A11 on these interactions in NRK-E52 cells we pre-incubated GST-EB1 on glutathione-agarose beads with saturating amounts of either 1A11 or the non-specific IgG molecule 4 U. After washes, the beads were used in precipitations from NRK-52E cell extracts followed by SDS-PAGE and Western blotting for CLIP-170, p150Glued and MCAK (Figure 1D). A summary of the antibody binding site and binding domains of the major EB1 interactants is shown in Figure S2.

Quantitative immunoblotting for full-length APC is difficult, so we assessed this interaction using extracts from cells transfected with a plasmid driving the expression of the APC-derived protein GFP-APC-C3 followed by Western blotting for GFP (Figure 1E). GFP-APC-C3 includes both the EB1-binding region and the basic MT-binding region in APC [11]. These experiments indicated that 1A11 binding inhibited the GST-EB1 interaction with p150Glued, had no detectable effect on the association with MCAK and increased the amounts of both CLIP-170 and GFP-APC-C3 precipitated by the fusion protein. Finally, we noted that endogenous EB1 was not displaced from MT ends in interphase cells microinjected with 1A11, suggesting that antibody binding did not inhibit the EB1 interaction with MTs (Figure 1F). We therefore conclude that 1A11 is not a function-blocking reagent, but that it perturbs the normal equilibrium between EB1 and its various binding partners.

The anti-APC antibodies used in this study were obtained from the CRUK RMAS hybridoma bank. ALI 12–28 recognises an epitope at the N-terminus of APC and has been characterised and used in other studies [46]. Both of the other antibodies used were raised against an immunogen consisting of a C-terminal APC fragment fused to MBP. We further defined the epitopes recognised by these antibodies by immunostainining cells transfected with a series of APC C-terminal fragments fused to GFP [11], [47]. The epitope recognised by antibody C-APC 28.9 was not present in any of these constructs though it did recognise full-length GFP-APC (not shown), indicating that although it lies towards the C-terminus of the protein it must be located upstream of aa2127. The epitope recognised by C-APC 9.9 was found to lie in the basic domain of APC, between aa2126–2511. This suggested that it might influence the APC interaction with MTs or other proteins that bind near the APC C-terminus.

To investigate this we first examined the APC-EB1 interaction. Extracts were prepared from cells expressing APC-C3-GFP. These extracts were incubated with either 4 U or C-APC 9.9 and precipitations performed with bead-bound GST-EB1. Precipitates were subjected to SDS-PAGE and examined for the presence of APC-C3-GFP. This experiment indicated that the binding of C-APC 9.9 to APC-C3-GFP in cell extracts potently enhanced the ability of GST-EB1 to precipitate the fusion protein. Therefore, with respect to EB1 binding C-APC 9.9 is not a function-blocking antibody but it appears to shift the normal binding equilibrium between APC and EB1.

### Most microinjected cells complete mitosis

Studies on the role of APC and EB1 during mitosis in mammalian cells have so far relied on siRNA technology [30], [35], [36]. Although these are well-executed and informative studies, the use of siRNA-mediated expression knockdown has a number of potential drawbacks. First, it is not possible to be completely confident about the level of knockdown achieved at the level of an individual cell under direct observation. Second, knockdown inevitably takes time. This potentially allows cells to compensate for any progressive loss of function. In addition and of particular relevance to the present study, phenotypic endpoints seen in mitotic cells might have been caused by the blockade of interphase processes rather than the inhibition of truly mitosis-specific events. Finally, it is difficult to conclusively exclude off-target effects in siRNA work. We therefore decided to use antibody microinjection into living NRK-52E cells and time lapse imaging to study potential roles for APC and EB1 during mitosis. This cell line maintains a flattened morphology during mitosis, facilitating microinjection and imaging of the mitotic apparatus and chromosomes. To ensure the specific targeting of mitotic events, prophase cells were identified and injected before nuclear envelope breakdown (NEB).

**Figure 2:**
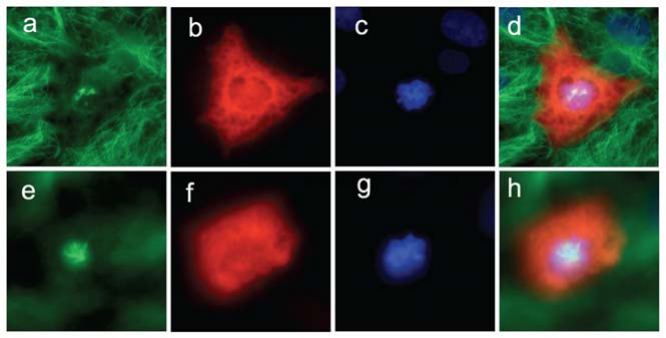
Spindle morphology in failed mitoses. Representative examples of cells which failed to complete mitosis following injection with either 4 U (panels a–d) or 1A11 (panels e–h). Fluorescently labelled dextran (red; panels b, d, f and h) indicates a successful microinjection. Immunostaining with an anti-tubulin antibody (green; panels a, d, e and h) reveals that these cells do not contain a properly organised mitotic spindle. Condensed DNA is revealed by the addition of DAPI (blue; panels c, d and g, h) Panels d and h show the merged images. Scale bar = 5 µM.

**Figure 3:**
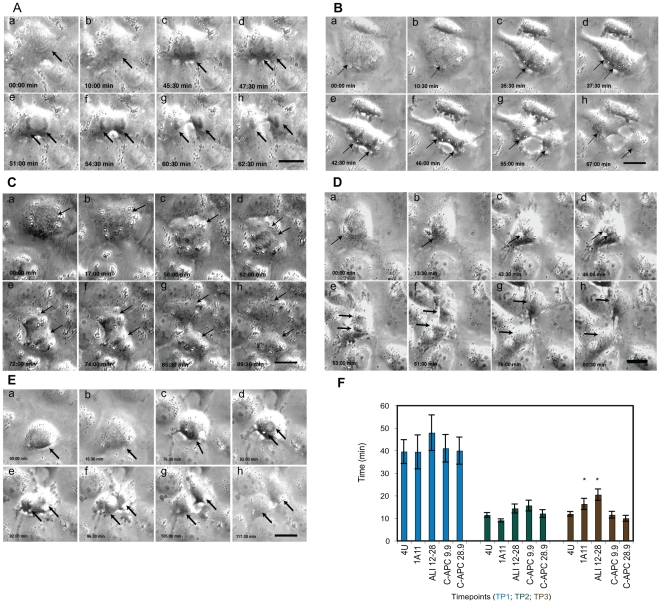
Mitotic progression in microinjected cells. Stills from representative time-lapse movies of mitotic NRK-52E cells microinjected with the five different monoclonal antibodies used in this study. A: cell microinjected with the non-specific control IgG 4 U. B: cell microinjected with the EB1 specific antibody 1A11. C: cell microinjected with the APC N-terminus specific antibody ALI 12–28, D: cell microinjected with the APC basic domain specific antibody C-APC 9.9. E: cell microinjected with the APC C-terminus specific antibody C-APC 28.9. Scale bar = 10 µM. a-g stills show different stages of mitosis, a- prophase, immediately after microinjection, b- prophase, c- metaphase, d- early anaphase, e- telophase, f–h- telophase/cytokinesis. The arrows indicate the position of the chromosomes. F. The time spent by injected cells in different stages of mitosis is summarised in this graph. Time point 1 (TP1) represents the time taken from nuclear envelope breakdown to anaphase onset. TP2 represents the time taken from anaphase onset to the start of telophase. TP3 corresponds to the time taken from the start of telophase until the appearance of a phase-dense midbody during cytokinesis. No significant differences in the duration of these time points was seen in cells injected with the different antibodies until TP3, which cells injected with 1A11 (p<0.05) and ALI 12–28 (p<0.005) took significantly longer to transit. The graph shows the mean time in min +/− SEM for each condition. Significant results are indicated with an asterisk. (4 U, n = 12; 1A11, n = 9; ALI12–28, n = 9; C-APC 9.9, n = 14; C-APC 28.9, n = 11).

The four specific monoclonal antibodies (one against EB1 and three against APC) described above and an unrelated control monoclonal antibody (4 U) were used for microinjection. The three APC-specific antibodies provide useful coverage of this large multi-domain protein. Immediately after antibody injection, cells were continuously monitored for at least 2 h by time-lapse microscopy. This time limit was chosen to avoid open-ended experimental durations and because uninjected cells transited mitosis well within this time (data not shown). First, we established whether microinjection with control antibody had a detrimental effect on mitotic cells. The rates of mitotic failure following microinjection were calculated and expressed as percentages (Table 1). 75% of the cells microinjected with the control antibody 4 U successfully completed mitosis within 2 h. Similar results were obtained for cells microinjected with the APC antibodies C-APC 9.9 (88.9%) and C-APC 28.9 (73.3%). However, there was a higher rate of failed mitosis in cells microinjected with the EB1 antibody 1A11 (58.9% completed) and the APC antibody ALI 12–28 (56.3% completed; Table 1), but these increased rates did not achieve statistical significance. Immunostaining of cells that had failed mitosis were examined for obvious morphological defects. Images of injected cells showed that the spindle had not properly formed, but there were no differences in the appearance of arrested cells injected with different antibodies (cells injected with 4 U and 1A11 are shown in Figure 2). We therefore cannot conclude that the higher failed mitosis rate for the cells injected with 1A11 and ALI 12–28 was due to a specific effect of these antibodies. We next examined the movies obtained from cells that completed mitosis to reveal the effects of antibody injection on a variety of mitotic processes. Figure 3A–3E shows representative still images from movies of cells microinjected with the five different antibodies used in this work. Specific mitotic stages are shown (**for full movies see Movies S1, S2, S3, S4, S5**).

### Microinjection with EB1 or APC-specific antibodies delays the onset of cytokinesis

It has been reported that siRNA-mediated depletion of APC led to transient anaphase delay in human cells [36]. We investigated the effect of injecting APC and EB1 specific antibodies on the duration of specific mitotic phases by determining the time taken by microinjected cells to transit defined time points. Time period (TP) 1 ran from NEB until metaphase congression, TP2 from the onset of anaphase until the onset of telophase and TP3 from telophase onset until the appearance of a phase-dense midbody.

Similar to 4 U-injected control cells, none of the cells injected with specific antibodies spent a prolonged period in TP1 and TP2. For example, 4 U injected cells spent 39.5±5.3 min in TP1 while 1A11, ALI 12–28, C-APC 9.9 and C-APC 28.9 injected cells spent 39.4±7.5, 47.9±7.9, 41.0±6.1 and 39.9±6.0 min respectively in the same mitotic stage. In TP2 the lengths of time spent were 11.4±1.0, 9±0.7, 14.3±2.0, 15.6±2.4 and 12±1.7 min. However, cells microinjected with either 1A11 (16.3±2.5 min; significant, p<0.05, *t*-test) or ALI 12–28 (20.4±2.5 min; significant, p<0.005, *t*-test) spent more time in TP3 than cells microinjected with 4 U (11.9±1.0 min), C-APC 9.9 (11.5±1.1 min) and C-APC 28.9 (9.9±1.3 min). These results implicate EB1 and APC in late mitotic events and they are summarised in Figure 3F.

### EB1 and APC injected cells possess a subtly disordered metaphase plate

**Figure 4:**
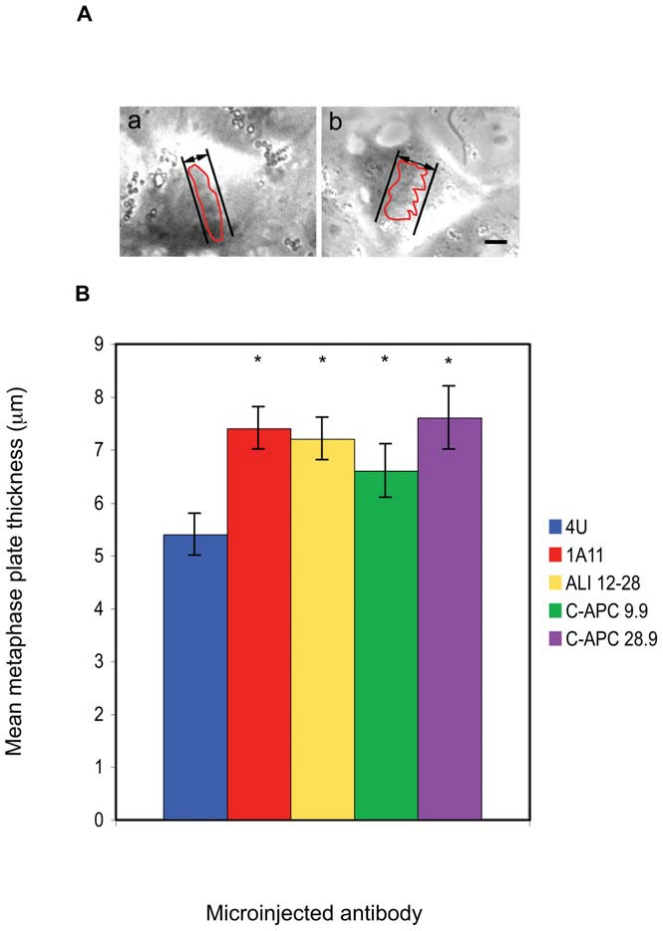
Microinjection with EB1 or APC specific antibodies affects chromosomal congression at the metaphase plate. A: Movies of microinjected cells were examined and the last frame before anaphase onset identified. The distance separating the widest two points of the metaphase plate was then measured as indicated. In the representative examples shown it can be seen that the metaphase plate was more tightly compacted in cells injected with control IgG (panel a) than in cells microinjected with 1A11 (panel b). Scale bar = 5 µM. The red traces indicate the outline of the metaphase plates. B: Summary of the metaphase plate thickness data. The metaphase plate was significantly wider in cells microinjected with APC or EB1 specific antibodies when compared to cells injected with the non-specific antibody 4 U. Mean distances +/− SEM are shown. Significant results are shown with an asterisk. P values were for 1A11, p<0.005; ALI 12–28, p<0.005; C-APC 9.9, p<0.05 and C-APC 28, p<0.005. (4 U, n = 11; 1A11, n = 14; ALI 12–28, n = 11, C-APC 9.9, n = 12; C-APC 28.9, n = 7).

siRNA experiments had previously shown that APC or EB1 depletion led to metaphase congression defects that included a disordered metaphase plate [36] and chromosomal misalignment [35]. We examined whether similar phenotypes were induced in microinjected cells. Our first observation was that in movies of control cells it was usually possible to anticipate the onset of anaphase. As this event approached, oscillations of the metaphase plate decreased while the plate itself became tightly compacted. However, in cells injected with all of the specific EB1 and APC antibodies we were unable to predict the imminent transition into anaphase. To objectively analyse this effect we measured the thickness of the metaphase plate across its widest point in the final frame before chromosomal segregation was initiated (Figure 4A). We determined that EB1 and APC antibody microinjected cells possessed a metaphase plate that was significantly wider than that present in 4 U injected cells. The average metaphase plate in 4 U microinjected cells was 5.4±0.3 µm in comparison to 7.4 µm±0.4 in 1A11 injected cells (significant, p<0.005, *t*-test), 7.2 µm±0.4 in ALI 12–28 injected cells (significant, p<0.005, *t*-test), 6.6 µm±0.5 in C-APC 9.9 injected cells (significant, p<0.05, *t*-test) and 7.6 µm±0.6 in C-APC 28.9 injected cells (significant, p<0.005, *t*-test) (Figure 4B). Metaphase plates in cells injected with EB1 and APC-specific antibodies were otherwise normal in terms of gross morphology, with no increased incidence of lagging chromosomes, no evidence of chromosomal decondensation and no other obvious defects during prometaphase seen in our dataset. Taken in conjunction with our observation that no increase in the duration of metaphase was seen in injected cells (Figure 3F), we suggest that our observations are in general agreement with that of previous siRNA studies, confirming by a different methodology that perturbation of EB1 or APC function in metaphase cells causes a subtle disorganisation in chromosomal congression at the metaphase plate without preventing the eventual completion of mitosis.

### APC or EB1-specific antibodies do not cause spindle misalignment relative to the longest axis of injected cells

**Figure 5:**
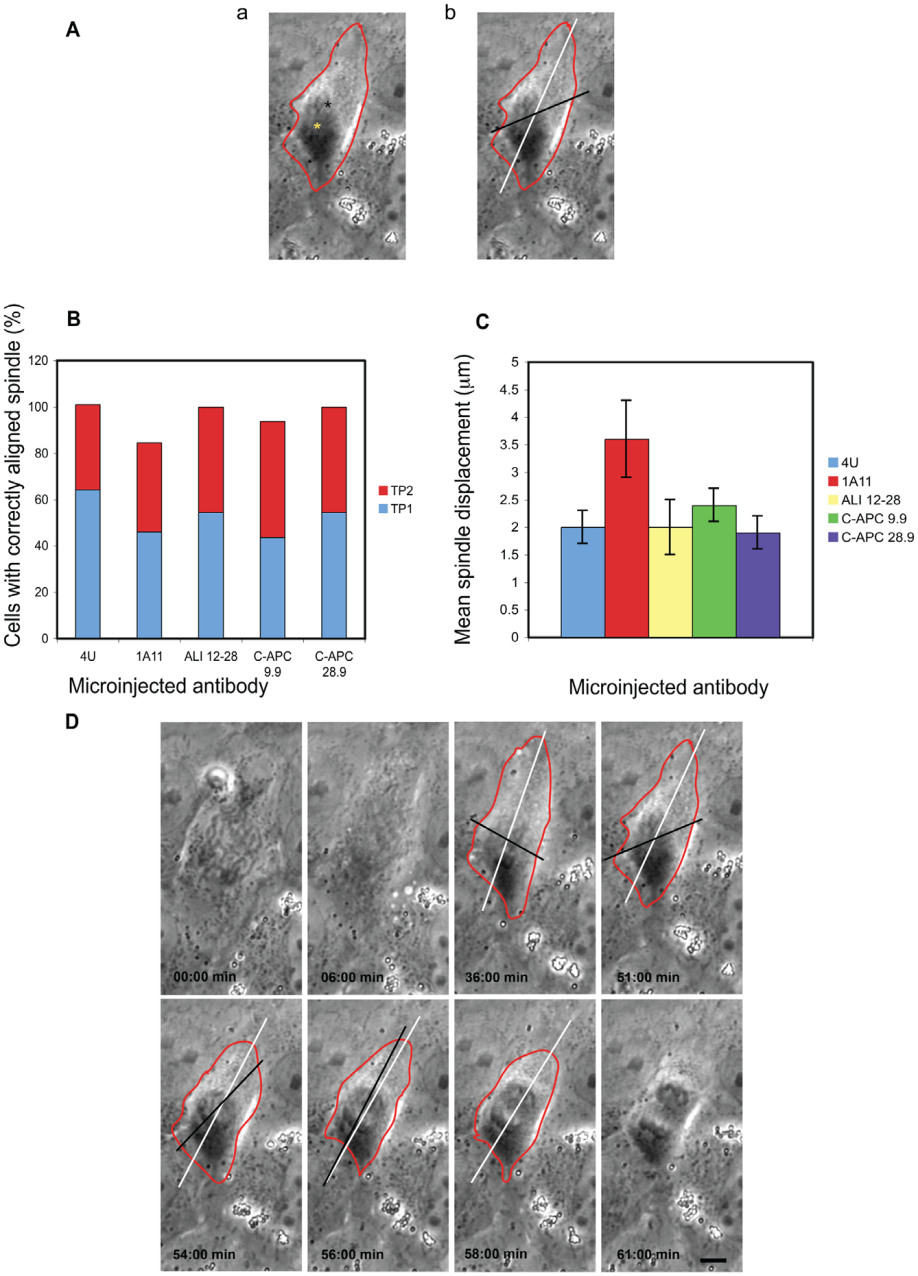
Effects of antibody injection on mitotic spindle positioning. Following antibody microinjection in prophase, spindle alignment and placement was assessed. A: Still image of a cell microinjected with 1A11 at metaphase displaying spindle displacement and misalignment, (a) the black asterix indicates the geometrical centre of the cell and the yellow asterix the centre of the metaphase plate; (b) the white line indicates the longitudinal axis of the cell and the black line the axis of the spindle. B: Spindle alignment in microinjected cells at TP1 (onset of anaphase) and TP2 (onset of telophase). At TP1 the greatest amount of spindle misalignment was observed among cells microinjected with 1A11 or C-APC 9.9 and these cells were also less likely to be correctly aligned at TP2. However, these differences did not achieve statistical significance. (4 U, n = 14; 1A11, n = 13, ALI 12–28, n = 11, C-APC 9.9, n = 9, C-APC 28.9, n = 11). C: Spindle placement within the mitotic cell at TP1 was unaffected by APC antibody microinjection, although spindles were displaced further from the geometric centre of the cell in cells injected with the EB1 specific antibody 1A11. However, this failed to reach statistical significance (p = 0.06). Mean spindle displacement +/− SEM is shown. (4 U, n = 11; 1A11, n = 14; ALI 12–28, n = 11, C-APC 9.9, n = 12; C-APC 28.9, n = 7). D: Movie stills taken from a recording of a representative cell microinjected with 1A11 and fixed at anaphase. Note that at metaphase (51:00 min) the spindle is misplaced and misaligned. Scale bar = 10 µm. For movie of these stills see Movie S6. The red outline shows cell shape, the white line indicates the longitudinal axis of the cell and the black line the axis of the spindle.

Effects on spindle alignment and placement after inhibition of EB1 and APC expression have been recorded [35], [36]. We therefore examined these events in our injected cells. Spindle alignment relative to the longest axis of the cell and spindle placement within the cell were measured as outlined in the Methods and Materials and examples are shown in Figure 5A.

We first observed that a higher proportion of cells microinjected with the EB1 antibody 1A11 and the APC antibody C-APC 9.9 were misaligned relative to the longest axis of the cell at the end of metaphase (Figure 5B), although these figures were not statistically significant. All misaligned spindles were correctly aligned by the end of TP2 except for a minority of spindles in 1A11-injected cells where only 84.6% were correctly aligned and in C-APC 9.9-injected cells where 93.7% were correctly aligned.

Only 1A11 appeared to have a detectable effect on spindle placement within the cell with an average distance of 3.6 µm±0.7 from the cell centre recorded. In comparison, spindles in cells injected with control antibody were on average 1.9 µm±0.3 from the cell centre. The greater misplacement seen in 1A11-injected cells was not significant (p = 0.06, *t*-test) (Figure 5C). However, it should be noted that cell size effectively places a limit on the maximum possible distance from the geometrical centre a spindle can be located, so the mean value obtained for 1A11 injected cells could be considered to be more significant than indicated by statistics. For similar reasons, the increase in spindle size relative to cell size during anaphase meant that the measurement of spindle placement in the later stages of mitosis was impractical using this methodology. An example of spindle misalignment relative to the longest axis of the cell and spindle misplacement can be seen in Figure 5D, which shows stills of a cell microinjected with 1A11.

### Microinjection with anti-EB1 mAb causes mitotic stage-specific membrane blebbing

**Figure 6:**
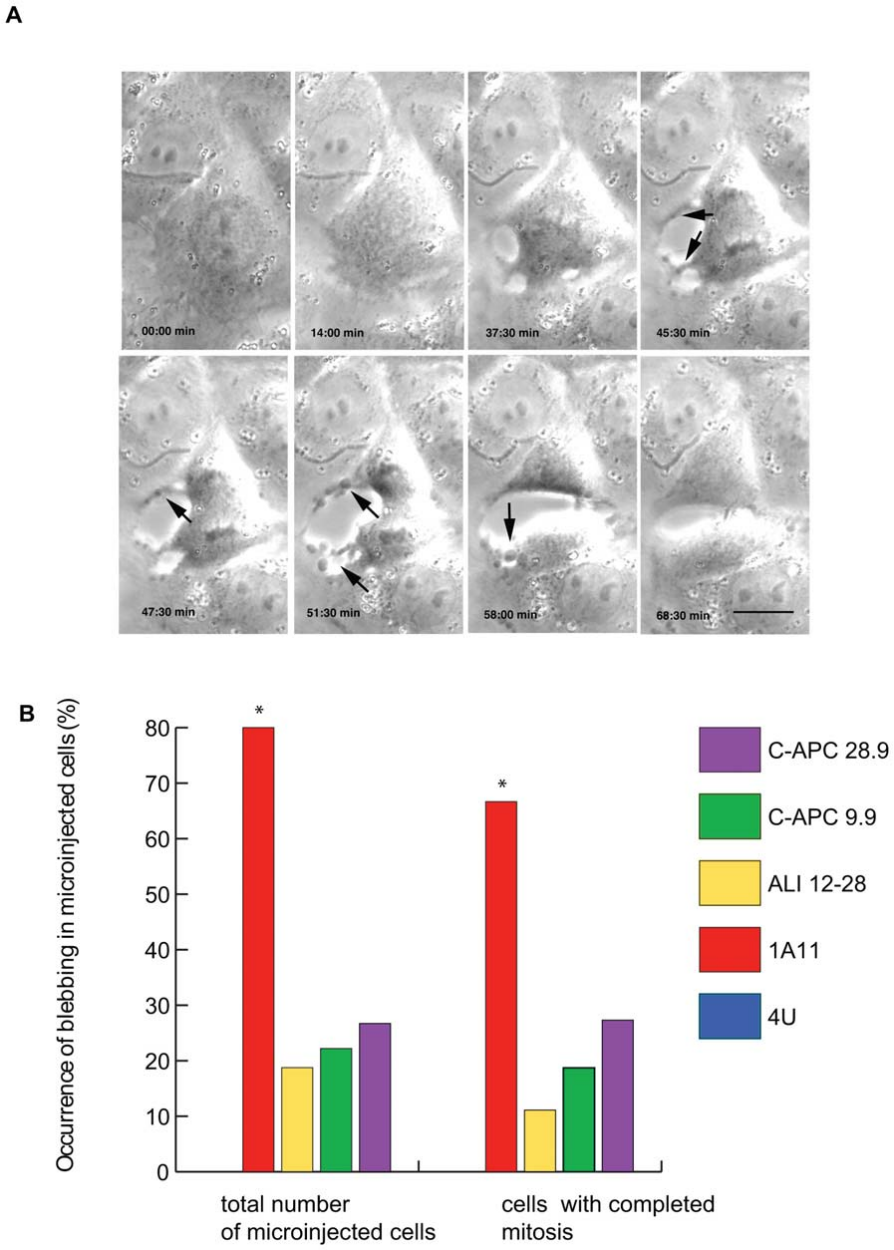
EB1 antibody injection induces anaphase-specific cortical blebbing. A: Stills from a recording of a representative cell microinjected with 1A11, showing the appearance of cortical blebbing during anaphase (arrows). Scale bar = 10 µM. B: The percentage of cells displaying cortical blebbing is first shown as a percentage of the total number of microinjected cells examined (including those that failed to complete mitosis) and then as a percentage of microinjected cells that successfully completed mitosis. In both cases a significantly higher proportion of cells microinjected with antibody 1A11 displayed blebbing (p<0.005 and p<0.05). 1A11-induced blebbing was only seen during anaphase whereas a lower level of blebbing was noted throughout mitosis in cells injected with anti-APC antibodies (see Figure 2 panels D and E for examples of blebbing in metaphase cells injected with anti-APC antibodies). Blebbing was not observed in mitotic cells injected with the non-specific IgG 4 U (zero entry in first column). Significant results are indicated by an asterisk. (4 U, n = 16 for total number of microinjected cells, n = 12 for cells with completed mitosis; 1A11, n = 19 and 11; ALI 12–28 n = 16 and 9; C-APC 9.9, n = 18 and 16; C-APC 28.9, n = 15 and 11).

During the analysis of the movies of injected cells, blebbing was observed in some of the cells microinjected with APC and EB1-specific antibodies (Figure 6A). In contrast, blebbing was not recorded in uninjected cells or in cells microinjected with 4 U. 66.7% of 1A11 injected cells displayed mitotic blebbing (p<0.05, Chi-square test), in comparison to 11.1% of ALI 12–28 injected cells, 18.7% of C-APC 9.9 injected cells and 27.3% of C-APC 28.9 injected cells (Figure 6B). When these cells were more closely analysed it was found that blebbing in the APC antibody injected cells typically arose during pro/prometaphase and persisted throughout mitosis. In contrast, the blebbing in cells injected with 1A11 was initiated at anaphase onset (Figure 6A, table S1). In all cases the observed blebbing subsided with mitotic exit. To investigate stage specific blebbing further we co-microinjected 1A11 and ALI 12–28 and recorded mitotic behaviour as before. Co-microinjection did not change the late mitotic blebbing observed after 1A11 injection alone (Figure S3C and D) indicating that EB1 may have a function in late mitosis independent of APC. However, the early mitotic blebbing observed after microinjection of ALI 12–28 was suppressed by 1A11 co-microinjection. Our observations suggest that perturbation of APC and EB1 function leads to abnormalities in cortical contractility in mitotic cells.

### Asymmetry in spindle pole movement in 1A11-microinjected cells

**Figure 7:**
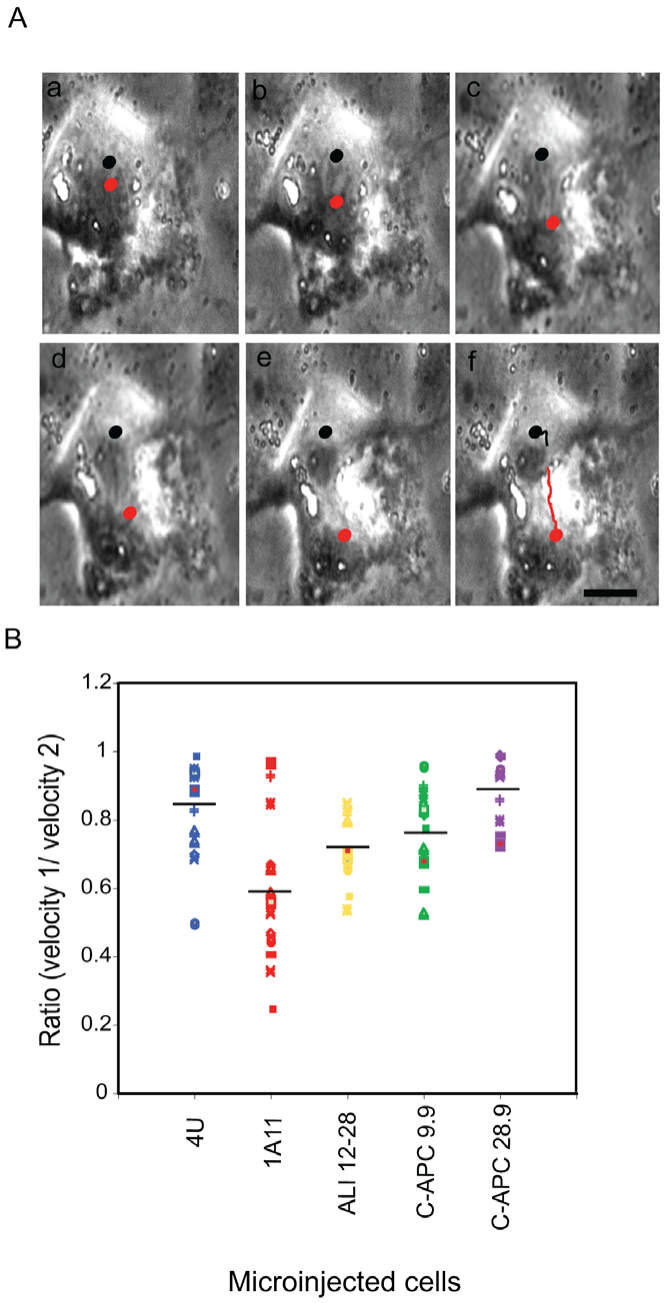
Microinjection of 1A11 induces asymmetric spindle pole movement. A: Stills (a–f) taken from a recording of a representative cell microinjected with 1A11. As shown by the tracks, one set of chromosomes (the black spot closest to the top of the frame) remains relatively static within the cell during anaphase B and telophase while the other moves. The black and red tracks indicate chromosomal movement. Scale bar = 10 µM. B: Velocities were obtained for anaphase chromosomal movement in microinjected cells and used to derive a ratio that reflected any asymmetry in the observed movements, with values close to 1 representing symmetrical chromosomal separation. A plot showing the range of velocity ratios obtained is shown. Only cells injected with the EB1 specific antibody 1A11 displayed an asymmetry in movement that generated a ratio of less than 0.5 (column 2). (4 U, n = 14; 1A11, n = 14; ALI 12–28, n = 10, C-APC 9.9, n = 12; C-APC 28.9, n = 9). Black line indicates mean value.

As part of our characterisation of the phenotype of injected cells we next examined spindle pole movement (Figure 7A). Movies were analysed with Tracking software, enabling us to measure spindle pole movements, using chromosomal movement as an easily trackable proxy, and express them as velocity values. The ratio of the velocities obtained for the two spindle poles in each cell were calculated, with figures closer to 1 indicating increased symmetry of pole movement. We were clearly able to observe chromosomal movements characteristic of anaphase A and B. We reasoned as the separation of the segregated chromosomes in anaphase B is known to be directly coupled to spindle pole movement during this phase of mitosis, that chromosomal movement is a fair reflector of spindle pole behaviour in this context. As shown in Figure 7B, chromosomal movement was more likely to be asymmetrical in cells injected with 1A11. Only among cells injected with this antibody was a ratio of <0.5 seen, indicating a significant difference in the movement of the spindle poles. This was recorded for 38.5% of the cells injected with 1A11, but it was not observed for any of the cells microinjected with either 4 U or any of the APC-specific mAbs. An example of uneven pole movement is shown in Figure 6A. Since initial anaphase chromosomal separation appeared to occur normally in injected cells, these observations are consistent with a role for EB1 in anaphase B spindle pole movement.

**Figure 8:**
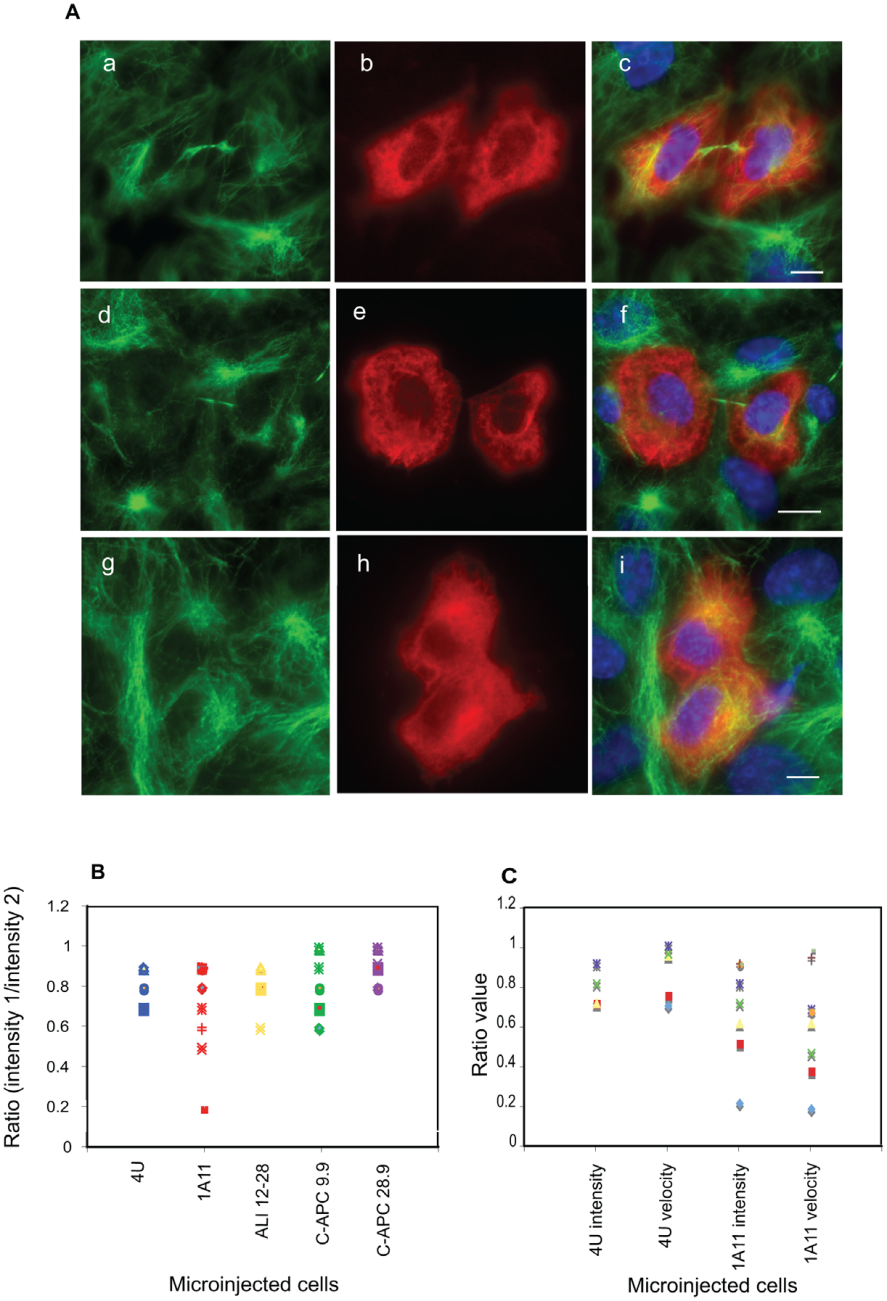
Asymmetrical microtubule content in daughters of cells injected with 1A11. Microinjected cells were allowed to complete mitosis then were fixed and immunostained to reveal the MT cytoskeleton. A: Images obtained from daughter cells after microinjection of mothers with 4 U and 1A11. Daughters of cells injected with the control antibody 4 U were equally sized and displayed an equal content of microtubules (a–c), whereas the daughters of 1A11 injected cells exhibited more uneven microtubule contents (d–i) and in some cases were of unequal sizes (d–f). Scale bar = 5 µM. B. The fluorescence content of daughter cells immunostained for tubulin was measured and used to derive a ratio that reflected any asymmetry in the MT content of the cells, with values close to 1 representing essentially equal MT contents. A plot showing the range of ratio values obtained is shown. The daughters of cells injected with 1A11 were more likely to exhibit asymmetry in MT content than cells injected with other antibodies. (4 U, n = 6; 1A11, n = 9; ALI 12–28, n = 5; C-APC 9.9, n = 6; C-APC 28.9, n = 7). C: A plot comparing the spindle pole movement and microtubule intensity ratios obtained for 4 U injected cells, n = 5 and 1A11 injected cells, n = 8). Mitoses where severely asymmetrical pole movement was seen also generated daughters with the most asymmetrical MT contents, with daughters inheriting the least mobile pole containing fewest MTs. Here, the same coloured shape indicates results for the same cell within either the 4u- microinjected group of cells or the 1A1-microinjected group of cells.

To investigate this phenotype further, the daughters of microinjected cells that had completed mitosis were fixed and immunostained for tubulin (Figure 8A). The MT content of these cells was calculated by measuring the fluorescence intensity of this staining and the two intensity values were compared. 1A11 injected cells were found to produce a higher proportion of daughter cell pairs with differing MT contents than the daughters of cells injected with the other antibodies (Figure 8B). It was also notable from this work that in some cases the induced asymmetry in pole movement led to the production of daughter cells of unequal size (Figure 8A, panels d–f). Comparison of immunostained cells with the corresponding time-lapse series indicated that the fewest MTs were seen in the cells that inherited the spindle pole that moved least during anaphase. Interestingly, the cells that had displayed the most asymmetric spindle pole movement also generated daughter cells with the greatest discrepancy in microtubule content (Figure 8C).

**Figure 9:**
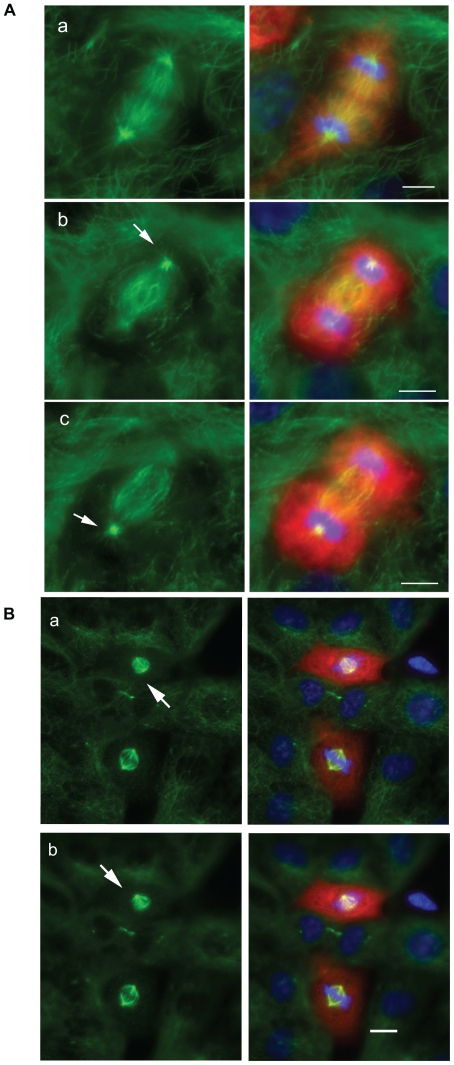
Microinjection of an anti-EB1 antibody induces tilted spindles. A. Immunostaining images of 4 U microinjected cells (a) and 1A11 (b,c) fixed and stained at anaphase. The images for the 1A11 injected cell had to be taken separately as the spindle poles were not in the same focal plane. Arrows indicate the two spindle poles, which were not in the same focal plane. Tubulin in green, combined anti-EB1 and dextran staining in red, DAPI in blue. Scale bar = 5 µM. B. Immunostaining image of 1A11 microinjected cells at metaphase. Arrow indicates a microinjected cell whose spindle poles are not in the same focal plane, revealing the presence of a spindle tilted in relation to the substrate. Tubulin in green, injected 1A11 and fluorescent dextran staining in red, DAPI in blue. Scale bar = 10 µM.

A further observation from this work was that in 1A11-injected cells the anaphase spindle poles were typically not in the same focal plane (Figure 9A). This observation was reproducible in cells microinjected with 1A11 then fixed and immunostained at metaphase (Figure 9B), where 70% of cells (n = 10) had spindle poles that were not in the same focal plane. In comparison, when control NRK-52E cells were fixed and stained, only 4% of metaphase cells (n = 100) showed the same phenomenon. We conclude from this that EB1 acts in pathways that maintain mitotic spindle orientation parallel with the substrate in NRK-52E cells, consistent with previous findings in a different experimental system [48]. This is likely to be a form of spindle misalignment mechanistically related to deviations in spindle positioning along the longest axis of dividing cells. Our failure to detect a statistically significant increase in the latter (Figure 5) may therefore represent limitations inherent in our experimental system.

### Spindle poles are unevenly decorated with EB1 at prophase

**Figure 10:**
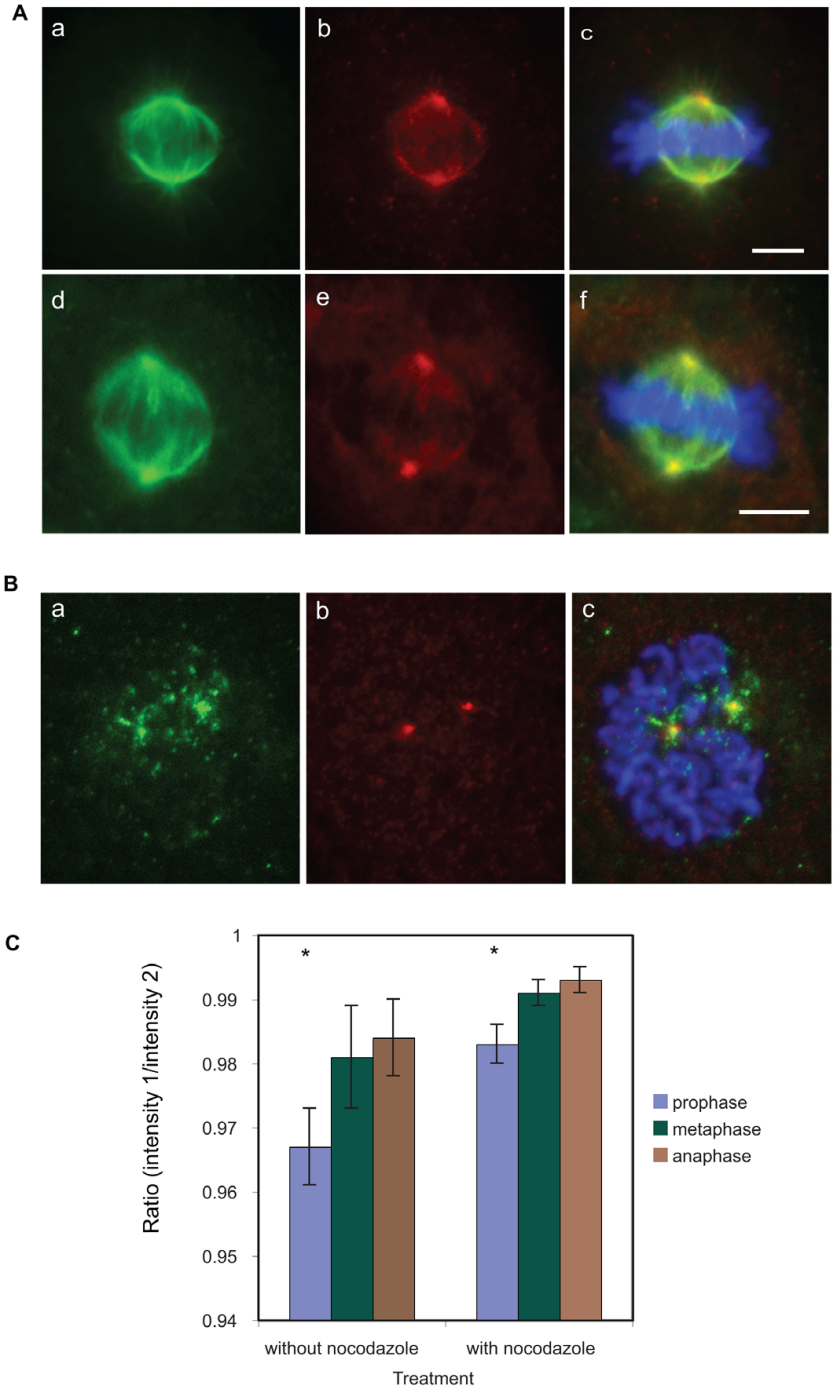
Characterisation of antibody 1A11 binding to EB1 in mitotic NRK-52E cells. A. Binding pattern of the EB1 specific antibody 1A11 in mitotic NRK-52E cells. The antibody binds to the spindle poles in fixed and immunostained cells (a–c) and microinjected, fixed and stained cells (d–f). Tubulin green, EB1 red, DAPI blue. Scale bar = 5 µM. B. EB1 staining patterns of spindle poles at prophase appear to be uneven as indicated by immunofluorescence of fixed and stained NRK-52E cells. EB1 green (panel a), centrin red (panel b), DAPI blue. Scale bar = 5 µM. C. EB1 is more unevenly distributed on centrosomes in prophase than in the later stages of mitosis, especially when compared to anaphase, in both the presence and absence of nocodazole (significant results are indicated with an asterisk, p<0.05).

It is known that EB1 localises to centrosomes and spindle poles and that it is involved in MT nucleation and/or anchoring at these sites [17], [49]. Strong spindle pole labelling was obtained when NRK-52E cells were fixed and immunostained with 1A11 or when cells injected with 1A11 were fixed and probed with an anti-mouse secondary antibody to reveal the binding location of the injected antibody, indicating that 1A11 binds strongly to EB1 at this site (Figure 10A). Western blotting of NRK-52E cell extracts confirmed that this signal was obtained by antibody binding to endogenous EB1 (Figure 1C). However, our imaging data suggested that in many injected cells the antibody had an asymmetric effect on spindle pole function. It has been reported that EB1 is present at the mother centrosome throughout the cell cycle but is only recruited to the daughter centrosome during the process of centrosomal maturation [49], though the precise timing of this recruitment is unclear. It therefore seemed possible that the injection of 1A11 into prophase cells might have occurred at a time when the recruitment of EB1 to the maturing daughter centrosome was incomplete. To test this we examined whether EB1 staining was uniform at both spindle poles in fixed and immunostained prophase, metaphase and anaphase cells. NRK-52E cells were co-stained with 1A11 and an anti-centrin antibody for independent identification of spindle pole location (Figure 10B). Quantitative analysis of fluorescence intensity revealed a subtle asymmetry in EB1 immunostaining at prophase centrosomes that became less obvious with progression through to anaphase. A significant difference was determined between the prophase mean and anaphase mean ratios of spindle pole staining intensity (p<0.05, *t*-test). This was not observed for centrin, but it was also evident when cells were immunostained with the EB1 antibody clone 5. Interestingly, treatment with nocodazole largely prevented this phenomenon while substantially decreasing the overall EB1 labelling at the spindle poles, suggesting that MTs played a role in the observed asymmetry (Figure 10C). Again, the mean values obtained for prophase and anaphase were significantly different in drug-treated cells (p<0.05, *t*-test).

### Microinjection of a dominant-negative EB1 protein confirms the data obtained by 1A11 injection

**Figure 11:**
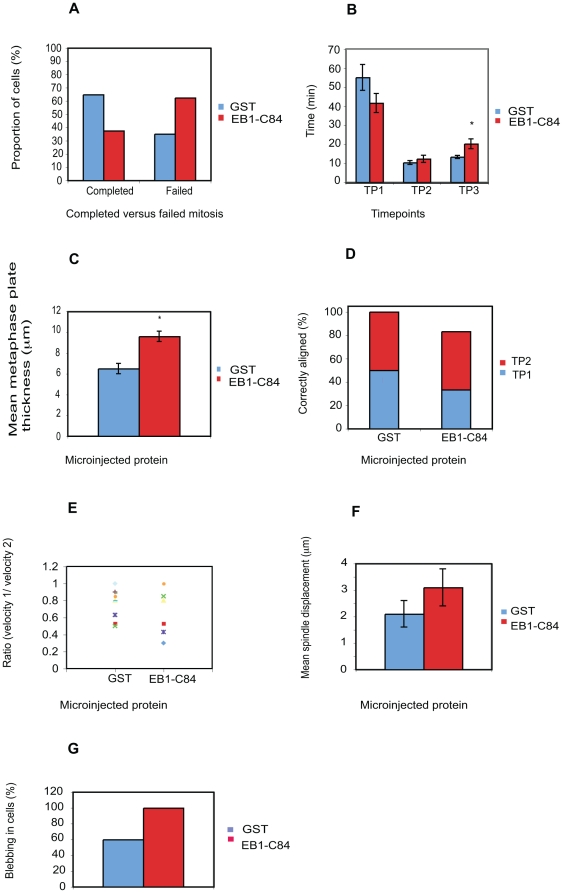
Microinjection of an inhibitory EB1 fragment into NRK-52E cells recapitulates phenotypes observed in EB1 antibody injected cells. Mitotic NRK-52E cells were microinjected with the dominant-negative EB1 fragment GST-EB1-C84 or with GST alone. A) Injection of low levels of GST-EB1-C84 inhibited progression through mitosis. B) Cytokinesis was significantly delayed in mitotic cells injected with GST-EB1-C84 that completed mitosis (p = 0.01). C) Chromosomal congression to the metaphase plate was significantly inhibited in cells microinjected with GST-EB1-C84. D) The majority of cells microinjected with GST-EB1-C84 had misaligned spindles at TP1, most of which had not corrected by the time cells reached telophase. E) Some cells microinjected with GST-EB1-C84 showed severe asymmetry in spindle movement, as shown by a ratio value of <0.5. Different colours and shapes represent individual cells. (F) Cells injected with GST-EB1-C84 tended to possess spindles that were placed further from the cell centre, though this trend did not reach statistical significance. (G) A higher proportion of cells injected with GST-EB1-C84 exhibited cortical blebbing, though it was not mitotic stage specific.

**Figure 12:**
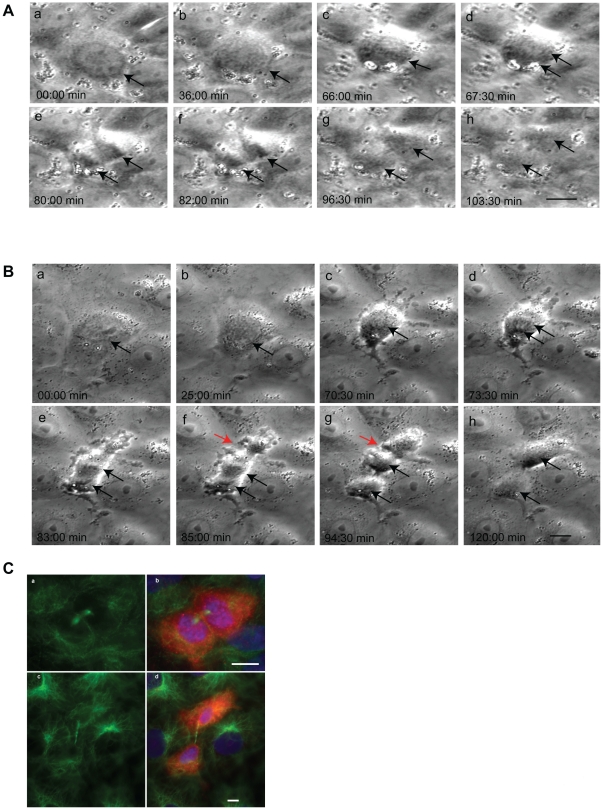
Microinjection of mitotic NRK-52E cells with GST or GST-EB1-C84 fusion protein. Stills from representative time-lapse movies of mitotic NRK-52E cells after microinjection with GST alone (A, a–h) or GST-EB1-C84 (B, a–h). Black arrows indicate the position of the chromosomes. Note cortical blebbing and the formation of an ectopic cleavage furrow, which is resolved by the time the GST-EB1-C84 injected cell reaches cytokinesis (B, red arrow) C: The cells shown in A and B were fixed and immunostained with an antibody specific for alpha-tubulin (green) and counterstained using DAPI (blue). Injected cells were identified by the presence of fluorescent dextran (red). The cells shown in a and b are daughter cells of the cell injected with GST, while the cells in c and d are daughter cells of the cell injected with GST-EB1-C84. Note the formation of uneven daughter cells after microinjection with the GST-EB1-C84 protein. Merged images are shown in b and d. Scale bars = 10 and 5 µm.

Although the experiments utilising the injection of antibody 1A11 were properly controlled and revealed phenotypes not seen using a non-specific IgG, we wished to further confirm that the effects seen were specifically caused by perturbation of EB1 function in mitotic cells. We have previously described the dominant-negative effects of an EB1 C-terminal fragment termed EB1-C84 when it was expressed as a GFP fusion protein in transfected cells [17]. We therefore produced a purified recombinant form of this protein, GST-EB1-C84, and used both this reagent and purified GST in a further series of microinjections. Initially, both proteins were used at a needle concentration of 1 mg/ml. However, although the majority of GST-injected cells successfully completed mitosis all cells injected with GST-EB1-C84 (n = 10) arrested in mitosis having failed to assemble a spindle (data not shown). This phenotype is consistent with the complete inhibition of EB1 function in injected cells, potentially combined with the dominant-negative functional inhibition of EB1 ligands. For example, GST-EB1-C84 binding to p150Glued might be expected to at least block the direct interaction of this protein with MTs [50] and the consequences of inhibiting dynactin function in mitotic cells are well documented [51]. This contrasts, of course, with the more subtle inhibition of normal EB1 activity expected to occur in 1A11-injected cells. Therefore, in an attempt to achieve a less severe inhibition of EB1 function using this reagent we reduced the needle concentrations of the purified proteins to 100 µg/ml. Under these conditions 37.5% of cells with GST-EB1-C84 successfully completed mitosis (Figure 11A) in comparison to 64.8% of GST only injected cells. Interestingly, GST-EB1-C84 injected cells displayed several phenotypes which mirrored those observed in EB1 antibody injected cells. For example, a delay at TP3 (p = 0.01, *t*-test, significant), diffuse metaphase plates (p<0.001, *t*-test, significant), a high proportion of misaligned spindles relative to the longest axis of the cell (66.6%) that had not aligned by TP2 (12.1%), and severe asymmetric spindle pole movements were observed (Figure 11B–E). Again, displacement was not significantly different (Figure 11F). Blebbing was recorded in all injected cells in comparison to 60% of control injected cells, although it did not seem to be stage specific (Figure 11G). It is possible that microinjection of GST only at the concentration we used induced blebbing as a cellular response to injection with this particular protein; however, in contrast blebbing was seen in *all* the cells microinjected with GST-EB1-C84 suggesting that there is also association of EB1 with cortical contractility even though we did not record stage specific blebbing possibly masked by the GST only induced blebbing. Examples of control and EB1-C84 GST fusion protein injected cells are shown in Figure 12. Further investigation could include a tailless version of the EB1-terminus, as it cannot bind to most of the known partners of EB1 but acts as a dominant negative on EB1 itself [50].

We conclude from this work that most of the effects seen following microinjection with 1A11 are directly related to the perturbation of normal EB1 function in mitotic cells (**for full movie see Movies S7 and S8**).

## Discussion

**Figure 13:**
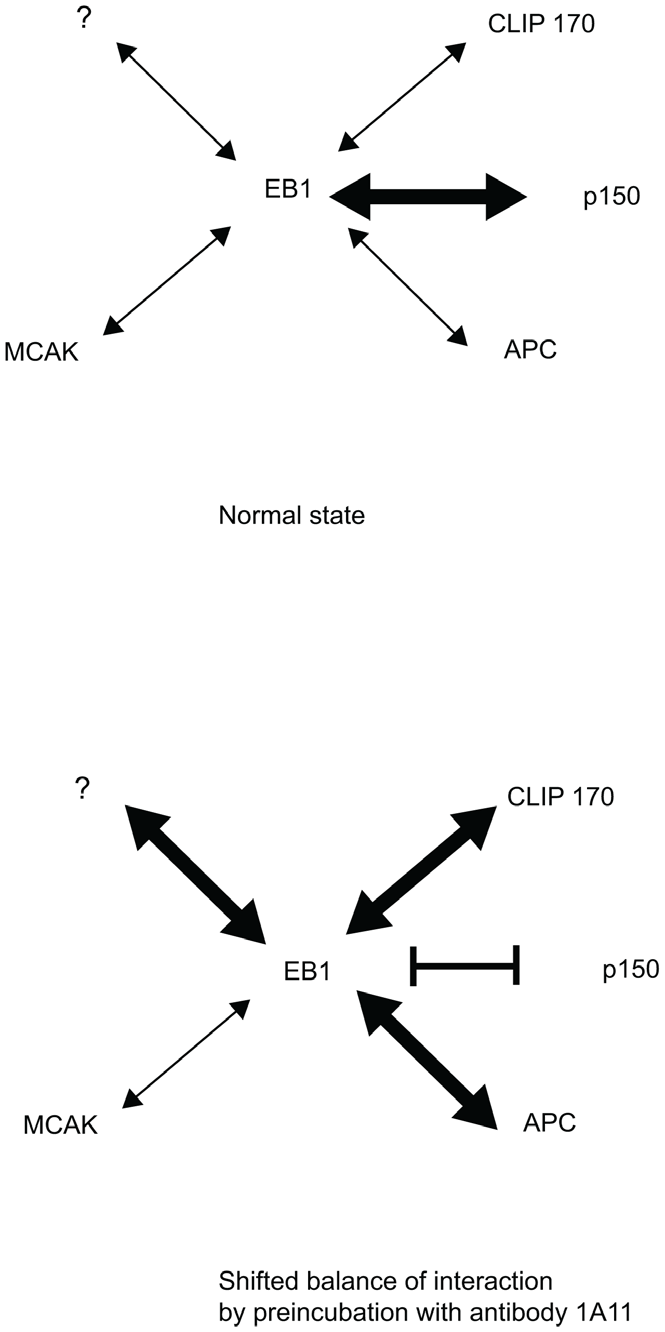
Diagram showing the possible interactions of EB1 with main interactants and the effect of antibody binding. Blocking of EB1 with 1A11 enhances some interactions (CLIP-170, APC), reduces interactions with p150Glued and has no effect on other interactions, for example with MCAK (shifted balance after preincubation with specific antibody).

In this study we aimed to investigate the roles of APC and EB1 in mitotic mammalian cells by antibody microinjection. Our antibody characterisation suggested that rather than directly inhibiting EB1 or APC function the antibodies used induced a shift in the usual balance of ligand binding, which in turn influenced EB1 and APC function. Perhaps the most likely explanation for the EB1 data is that p150Glued appears to be the most important EB1 ligand within cells, as evidenced by findings in a previous study where GST-EB1 was used in pull-downs from extracts of cells containing radiolabelled proteins [52]. The major precipitated species observed in this experiment were p150Glued and its binding partner cytoplasmic dynein intermediate chain. Since we have previously shown that EB1 ligand binding is competitive [17], a simple interpretation of our findings is that a reduction in p150Glued binding permits increased interactions with CLIP-170 and APC, which are key players in MT interactions and which may have an effect on MT dynamics (Figure 13). Additionally, it is well documented that p150Glued interaction with EB1 is instrumental in anchoring newly nucleated microtubules at centrosomes and perhaps also at the spindle poles during mitosis [17], [53], [54]. Reducing p150Glued interactions with EB1 in the earliest stages of mitosis via antibody blocking might therefore reduce the efficiency of microtubule anchoring at spindle poles, potentially explaining the phenotypes observed in our experiments.

Recent findings on the roles of APC and EB1 in mitosis have suggested that they act during chromosomal congression and segregation and are involved in spindle orientation, functions that are likely to be mediated by their interactions with MTs. We therefore examined our data for phenotypes associated with these processes. Our analyses revealed that there were some phenotypes associated with the injection of both APC and EB1 antibodies, implying that both proteins act in a common pathway, while some were specific for the EB1 antibody, suggesting that it has mitotic roles independent of its interaction with APC. In the first group, our results confirmed data recently presented by other workers [35], [36]. We found that injection of antibodies against APC and EB1 induced a less organised metaphase plate without preventing the eventual completion of mitosis. We also noted effects on the spindle itself after antibody microinjection. Injection with 1A11 also increased the incidence of spindles that were tilted in relation to the cell substrate (Figure 9), a phenotype that can be considered to be a form of spindle misalignment and that again confirms that an EB1 phenotype first identified by a siRNA approach is recapitulated by our methodology [48]. However, we were unable to identify a significant role for EB1 in spindle alignment along the longest axis of dividing cells, a phenomenon that one would assume was mechanistically related to our observation of tilted spindles. This probably represents a consequence of limitations in our experimental system, most likely related to our small sample size since a trend of misalignment was seen that failed to reach significance. We were also unable to confirm that APC plays a role in spindle alignment or placement in our experimental system.

We were therefore able to induce by microinjection at prophase a number of phenotypes previously observed by other workers following the knockdown of EB1 or APC expression. This indicates that these effects are directly associated with the function of APC and EB1 during metaphase and that they are not a consequence of an interphase defect affecting subsequent mitoses, or a result of cell adaptation in response to a gradual depletion of protein levels. They also indicate that our experimental approach is valid and that the injected mAbs are affecting the function of their targets. In the case of C-APC 9.9, epitope mapping suggests that this could be due to a direct inhibition of the APC interaction with MTs. Intriguingly, our data also revealed two striking phenotypes that were specifically associated with EB1 antibody-microinjection but had not been reported in previous studies. These were anaphase-specific cortical blebbing and asymmetric spindle pole movement.

### EB1 antibody injection causes anaphase-specific cortical blebbing

Movies of microinjected cells revealed that cells injected with 1A11 often displayed cortical blebbing during anaphase. Blebbing is not uncommon in mitotic cells. However, in our hands cortical blebbing during mitosis was never seen in either uninjected NRK-52E cells or cells injected with control IgG. Furthermore, it is unlikely that the blebbing observed after mAb injection was associated with apoptotic processes since it did not lead to cell death and resolved in daughter cells when mitosis was completed.

Blebbing was also seen at a much lower level in cells injected with the anti-APC mAbs. However, this blebbing was observed from prophase onwards and did not display any specificity for a particular mitotic stage. It is known that APC interacts with the actin cytoskeleton in migrating cells via factors such as the Cdc42-activating factor Asef and IQGAP1 [55]–[57] while we have examined its actin-dependent localisation to adhesive membranes [46] and noted a cortical localisation in mitotic NRK-52E cells [58]. It therefore seems possible that the blebbing observed after microinjection with the APC antibodies may be due to the disruption of an APC function that impacts upon the actin cytoskeleton in mitotic cells but is independent of its interaction with EB1.

The blebbing induced by 1A11 is arguably of greater interest. Our data suggests that this phenotype is caused by perturbation of an EB1-specific function in the late mitotic cell that is independent of its interaction with APC. It is tempting to speculate that this function is involved in the correct specification of the cleavage furrow. It is now clear that spindle MTs, in particular astral MTs and those forming the spindle midzone [59]–[61], play an important role in controlling cortical contractility. This is achieved, at least in part, through the local activation of RhoA. EB1 is known to interact with the Rho effector mDia [62], while in *Drosophila* an interaction with a Rho activating protein (DRhoGEF2) has been identified [18]. If a similar interaction exists in mammalian cells EB1 would be well placed to fulfil a role in the MT-dependent regulation of contractility at the mitotic cell cortex. An alternative explanation would be that EB1 function is involved in repressing ectopic cleavage furrows or contributing to cortical relaxation away from the cleavage furrow. Severe blebbing was observed in *Dictyostelium* and *Drosophila* after loss of the actin nucleation factor SCAR/WAVE suggesting a role in cell cortex maintenance. Depletion of EB1 revealed a role in localising SCAR at mitosis as well as migration [63]. This role appears to be recapitulated in mouse melanoma cells where EB1-depletion leads to the inhibition of lamellae formation and migration [64]. However, our previous work [65] has shown an increase in astral microtubule length and number in anaphase NRK-52E cells and images of microinjected anaphase cells stained with another EB1 antibody in this study clearly show that astral microtubules in microinjected cells still reach the cell cortex (figure not shown).

To further investigate the blebbing phenomena we co-microinjected 1A11 with the APC antibody ALI 12–28. The simplest result to summarise from this work is that ALI 12–28 co-injection had little effect on the anaphase blebbing caused by 1A11 injection; this supports the contention that this blebbing arises from the perturbation of an APC-independent function for EB1, possibly by inhibiting the binding of p150Glued and/or promoting the interaction of CLIP-170 with EB1. On the other hand, we observed that co-injection of 1A11 suppressed the prophase-specific blebbing caused by ALI 12–28 injection. This is a more difficult result to explain. It seems likely that ALI 12–28 is an APC function-blocking reagent in this pathway. We also deduce from our antibody characterization work (Figure 1) that 1A11 promotes the EB1-APC interaction. 1A11 injection does not induce prophase blebbing, hence promoting the APC-EB1 interaction does not seem to impact on this APC function. However, our previous work would imply that 1A11 could have no effect on APC function at this time point, since the APC-EB1 interaction is blocked in early mitosis by APC phosphorylation [11]. It therefore remains unclear how 1A11 suppresses the effects of ALI 12–28 injection. We might speculate that ALI 12–28 effects on APC include an inhibition of APC mitotic phosphorylation that permits 1A11-promoted EB1 binding that somehow restores APC prophase function. Alternatively, and perhaps more likely, suppression of the EB1-p150Glued interaction or promotion of the EB1-CLIP-170 interaction might antagonize the effects of ALI 12–28 injection via pathways that are independent of APC, perhaps involving modulation of microtubule dynamics.

In all other parameters examined no additive or suppressive effects were seen when both 1A11 and ALI 12–28 were co-injected, suggesting that EB1 and APC act in the same pathway where these phenomena were seen with injection of each antibody alone (for example failure to complete mitosis, mitotic timings and spindle alignment; Figure S3A, B and E) and confirming that the effect of 1A11 on spindle pole movement was independent of APC (Figure S3F).

### A role for EB1 in spindle pole movement

In a proportion of cells injected with 1A11 the spindle poles were observed to move asymmetrically, with one pole essentially immobile in the most extreme examples. An analysis of the MT content in the two daughter cells of completed mitoses revealed that cells containing a centrosome derived from the less mobile pole contained fewer MTs than their siblings. Tellingly, the greatest discrepancy in MT content was found between cells produced by mitoses that displayed the most severe spindle pole movement defect. We have previously shown that a sharp increase in the length and number of astral MTs at both spindle poles occurs in anaphase NRK-52E cells and that this is required for efficient spindle orientation [65]. Spindle pole movement in these cells is mediated, at least in part, by pulling forces exerted on astral MTs by dynein/dynactin complexes [66]. In sum, these observations are consistent with the idea that injection of 1A11 specifically antagonises EB1 function at one spindle pole. As a consequence, astral MT nucleation and/or growth are impaired from this pole, preventing linkage between pole and cortex and denying the possibility of movement generated by MT motors. These phenomena may be explained by an extension of the known centrosomal functions of EB1 to mitotic cells [10].

The morphology and life cycle of the centrosome provides clues that could help clarify our observations [67]. During DNA replication in S-phase the centrosome duplicates in preparation for mitosis. A single centrosome consists of two centrioles, with the older termed the mother and the younger the daughter. The original centrosome duplicates by a semi-conservative mechanism, resulting in one new centrosome obtaining the older mother centriole and the other the daughter. The latter gradually matures by recruiting proteins that form a pericentriolar matrix that drives MT nucleation and, after mitosis, acquiring structures termed subdistal appendages that function in MT anchoring. During the entry to mitosis both centrosomes undergo a dramatic reorganisation, recruiting a number of accessory proteins as a result of which a step-change in MT nucleation capacity is achieved [67]. Could subtle differences in this process between the two centrosomes explain the phenotype encountered in 1A11-injected cells?

**Figure 14:**
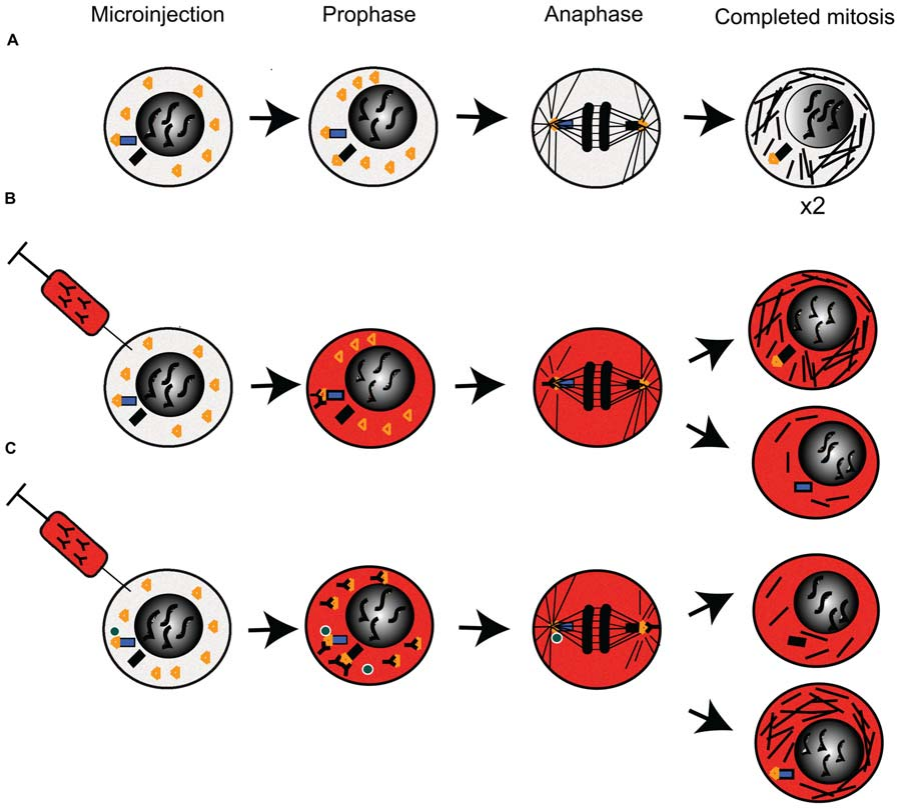
Models for the possible functional effects of antibody 1A11 in microinjected cells. (A) In uninjected cells EB1 (orange triangle) is initially localised to the mother spindle pole (blue rectangle) and is recruited to the daughter (black rectangle) during prophase, giving both poles functional equivalency in MT nucleation and/or anchoring during and after mitosis. (B) In microinjected cells the antibody binds preferentially to EB1 located at the mother spindle pole early in prophase and antagonises its function. The function of EB1 newly recruited to the daughter pole is not affected, leading to proper nucleation and/or anchoring of microtubules at the daughter but not the mother pole. The uneven distribution of functional EB1 at the poles leads to asymmetries in mitotic pole movement and MT content in daughter cells. (C) EB1 at the mother pole is associated with a binding partner (green circle) important for its function at this site. Antibody binding does not antagonise the function of pre-existing complexes, but it inhibits the functional associations of EB1 newly recruited to the daughter pole. This results in normal nucleation and/or anchoring of microtubules at the mother but not the daughter pole, again leading to asymmetries in mitotic pole movement and MT content in daughter cells.

Louie et al. [49] previously demonstrated that EB1 preferentially localises to the mother centrosome until the approach to mitosis. We propose that this asymmetrical association of EB1 with duplicated centrosomes, or the asymmetrical distribution of an EB1 functional interaction with a centrosomal binding partner such as p150Glued, could explain our results (Figure 14). In some early prophase cells functionally active EB1, regulated in an as yet un-identified way, could be primarily localised to the spindle pole containing the mother centriole. It seems possible that in some cells injection might have occurred early enough to block the function of EB1 at this older spindle pole but that EB1 subsequently recruited to the younger pole after injection was unaffected (Figure 14, model A). In this model the mother spindle pole is functionally impaired. In a more complex model, we can imagine a situation where at the point of antibody injection EB1 is already stably associated with a specific binding partner at the mother centrosome. In contrast, antibody-bound EB1 subsequently recruited to the daughter centrosome during prophase is unable to associate with this ligand. This model leads to a deficiency in an EB1 functional interaction at the daughter spindle pole during mitosis. In both models, the resulting imbalance in the amount of functional EB1 at the spindle poles could lead to uneven pole movement in anaphase by interfering with astral MT nucleation and growth at one pole in a manner analogous to that previously described in interphase cells when EB1 function at the centrosome is perturbed [17], [49].

Supporting the initial requirement for both of these hypotheses, we observed that prophase cells had a more uneven distribution of EB1 at the spindle poles, an asymmetry that normally disappeared as mitosis progressed. Furthermore, we identified target cells for injection on the basis of phase-visible DNA condensation but no NEB. This technique could not discriminate between cells in the very early stages of prophase and those where NEB was imminent. This might explain why only a fraction of microinjected cells showed severely asymmetrical spindle pole movement, since some cells may have been injected when EB1 functional recruitment to the daughter spindle pole was well advanced. It is also worth noting that this phenotype could easily have been missed in previous studies using siRNA-dependent knockdown of EB1 expression, since EB1 function at both poles would be likely to have been lost using this approach.

Further research on this topic will therefore be focused on investigating the function of EB1 and its potential binding partners at spindle poles in more detail. Observations from other experimental systems where EB1 proteins have been implicated in spindle asymmetries may provide useful cues in this work. For example, deletion of the fission yeast EB1 orthologue Mal3 in combination with the deletion of its binding partner Moe1 restricts the localisation of gamma-tubulin to only one spindle pole body and causes the formation of abnormal spindles [68], while in dividing *S. cerevisiae* the EB1 orthologue Bim1p complexed to Kar9p is initially restricted in its distribution to the older spindle pole body as part of a process that ensures proper spindle alignment [69]. Most recently, Fong et al. [70] demonstrated a direct interaction between EB1 and CDK5RAP2, a centrosomal protein, which was shown through its association with EB1 to localise to microtubules at the distal tips. By suppressing EB1 expression microtubule tip-tracking of CDK5RAP2 was inhibited leading to altered microtubule dynamics and stability.

In conclusion, we have demonstrated for the first time that microinjection of living mitotic mammalian cells with specific antibodies to APC or EB1 perturbs functions for these proteins in chromosomal congression and late mitotic events. Furthermore, EB1 appears to play a more widespread role in mitosis than previously revealed by siRNA studies, being involved in late mitotic processes that impact upon spindle pole movement and the regulation of cortical contractility. It seems likely that further research into the mitotic roles of EB1 and APC will lead to a better understanding of one of the most fundamental processes in cell biology.

## Methods

### Antibodies

Five monoclonal antibodies (mAbs) were used for microinjection in this study. These were a non-specific mouse IgG control, designated 4 U; 1A11, an EB1-specific mAb; C-APC 9.9, a mAb specific for the APC C-terminus; C-APC 28.9, another mAb raised against the APC C-terminus; and ALI 12–28, a mAb specific for the APC N-terminus. All of these antibodies were obtained as endotoxin-free preparations from the Cancer Research UK Research Monoclonal Antibody Service at concentrations ranging from 0.7–1 mg/ml in PBS. Antibodies used for immunostaining included a monoclonal rat anti-alpha tubulin antibody (Serotec), a rabbit polyclonal anti-centrin 1 antibody (Sigma) and the EB1-specific mAb clone 5 (BD Transduction Labs). All secondary antibodies were Alexa Fluor-conjugated reagents obtained from Molecular Probes.

### Cell culture

NRK-52E (normal rat kidney epithelial) cells obtained from the European Culture and Cell Type Collection were cultured as previously described [65]. Nocodazole treatment was performed essentially as described previously [8] with modifications introduced to maximise the suppression of MT growth without destroying the mitotic spindle, which would have prevented the identification of mitotic stages in drug-treated cells. Confluent NRK-52E cells were incubated with culture medium containing nocodazole at a concentration of 25 µg/ml for five min on ice. The cells were then immediately fixed and immunostained as described below.

### siRNA transfection of HeLa cells

HeLa cells were collected and resuspended in penicillin- and streptomycin-free growth medium and seeded into 6-well dishes (Costar), 1.6×10^5^ cells per well, for culture at 37°C, 5%CO_2_/95% air for 20–24 h. ON-TARGETplus SMARTpool siRNA oligonucleotides (Dharmacon) (sequences in table 2) were reconstituted in RNAse-free water (Ambion) to make 2 µM siRNA solution. This was mixed with DMEM and 2 µl DharmaFECT1 transfection reagent per well (Dharmacon) before dilution in penicillin- and streptomycin-free growth medium, according to the manufacturer's instructions, to make the siRNA transfection medium. Antibiotic-free growth medium was removed from the cultured HeLa cells and siRNA transfection medium was added prior to 48–72 h incubation at 37°C, 5%CO_2_/95% air.

**Table 2:** Sequence data for siRNA transfection.

Name	siRNA OligonucleotideSequences (5′-3′)	Target
	GGAAAGCUACGGAACAUUG	
ON-TARGETplus	AAACGACCCUGUAUUGCAG	EB1
SMARTpool MAPRE1	UGACAAAGAUCGAACAGUU	
	AGAUGAAGGCUUUGUGAUA	
	GAACAGGUACAUUCAUUAA	
ON-TARGETplus	GAAUGGCGGUCAAUGUGUA	EB2
SMARTpool MAPRE2	GAACGUUGAUAAGGUAAUU	
	GGAGUAUGAUCCUGUAGAG	
	CCUCAACUAUACCAAGAUA	
ON-TARGETplus	CAGCAAACUUCGUGACAUC	EB3
SMARTpool MAPRE3	GUAGAGAAAUUAGUGAAAG	
	UGAGACUGAUGCCCAAAUU	

Table 2 summarises the sequences for the ON-TARGETplusSMARTpool siRNA oligonucleotides used in the si RNA transfection.

### Microinjection

On the day of microinjection, the mAbs or EB1-C84 GST fusion protein and GST-protein only were prepared by adding Alexa 594-conjugated dextran to the antibody solution. They were then centrifuged at 4°C for 30 min at 13000 rpm and kept on ice until used. The medium was removed from the confluent NRK-52E cell cultures and replaced with CO_2_-independent medium (Gibco) supplemented with glutamine and 10% foetal calf serum. The cells were then transferred to the enclosed heated stage of a Zeiss Axiovert inverted microscope and allowed to equilibrate for 15 min. Phase contrast imaging was used to identify prophase cells and cells that showed no sign of nuclear envelope breakdown (NEB) were chosen for microinjection. Needles (Eppendorf FemtoTips I) were loaded with 3 µl of antibody solution and microinjection was performed manually using an Eppendorf InjectMan NI 2 and Eppendorf FemtoJet System. Injected cells were checked by fluorescence microscopy to confirm a successful injection. Microinjected cells were then examined by phase contrast time lapse imaging essentially as described previously [71]. Typically, images were obtained using 1×1 binning and exposure times of less than 250 ms/frame with a time-lapse interval of 30 s, for up to 2 h. All analyses were performed on original datasets composed of multi-image TIFF files. For presentation as movies, image series were saved as uncompressed AVI files then cropped, compressed and converted into QuickTime movies using Adobe ImageReady 7.

### Immunostaining

Cells were cultured on sterile coverglasses, processed for immunocytochemistry using cold methanol fixation as described previously [8] and imaged by fluorescence microscopy using a Zeiss Axiovert 200 inverted microscope coupled to an Orca II ER CCD camera (in conjunction with excitation/emission filtersets for DAPI, FITC and TRITC) controlled by AQM6 software (Kinetic Imaging, Nottingham, UK). Figures were assembled using Adobe Photoshop 7.

### Determination of metaphase plate thickness

Movies of injected cells were examined and anaphase onset identified. The last frame prior to anaphase onset was then selected. Two parallel lines were drawn on either side of the metaphase plate in this frame using Andor IQ image analysis software. Each line contacted the points furthest from the centre of the plate. The distance between these two lines was then measured. The mean value for the data for all analysed cells was calculated and plotted as a bar chart.

### Determination of spindle alignment and placement

For determination of metaphase spindle positioning the still images of metaphase cells obtained for metaphase plate thickness determination were used. To determine anaphase spindle position movies were examined until the first sign of cleavage furrow formation was seen. The frame prior to this was used for analysis. For determination of spindle alignment the longitudinal axis, drawn through the geometrical centre of the cell, was determined and outlined on the still image. The axis of the spindle, drawn through both spindle poles, was marked as a second line. The angle between the cell axis and spindle axis was determined and values above 20° were considered to be misaligned [65], [66]. For determination of metaphase spindle placement the geometric centre of the cell was determined. The central point of the metaphase plate was then defined and its distance from the cell centre measured. The mean value was determined for the values obtained for all the cells examined and plotted as a bar graph. We found that spindle placement could only be meaningfully examined in metaphase cells since anaphase spindle elongation forced essentially all spindles into a central position.

### Determination of anaphase spindle pole movement

Movies of injected cells were examined from the end of metaphase until telophase onset using Andor IQ Tracker software to follow the position of the centre-most point in each set of segregating chromosomes as a proxy for the position of the spindle poles, the positions of which were found to be difficult to unambiguously determine in phase contrast images. The values obtained for overall pole movement therefore also include a component derived from chromosomal separation in anaphase A, which appeared to occur normally in injected cells. Velocity values for pole movement were then determined. To reveal any asymmetries in spindle pole movement a ratio of the two velocity values was calculated. If the velocity of spindle pole movement was the same for both poles the ratio should be close to 1 for normal pole movement, whereas uneven pole movement would be highlighted by a value less than 1 with more severe cases nearing 0. The results were expressed as ratio values rather than actual traces of individual cells to highlight those cells with severe spindle pole movement.

### Analysis of fluorescence intensities

The fluorescence intensities of microtubule staining in daughter cells derived from microinjected mothers was determined as follows. First, cell areas were determined using the outline of the dextran fluorescence in the daughter cells as a guide. The total amount of tubulin fluorescence in each daughter cell was then determined using the Andor IQ software to obtain an integrated intensity value. This was divided by the area value to allow comparison of microtubule content in the two daughter cells.

The intensity of EB1 staining at spindle poles was determined by identifying the location of the poles using centrin immunostaining and then measuring EB1 fluorescence intensity at the poles using the Andor IQ software as described above.

### SDS-PAGE and Western blotting

Cells from confluent 25 cm^2^ flasks were extracted in lysis buffer (PBS, 1% (v/v) Triton X- 100), containing EDTA free complete protease inhibitor mix (Sigma) and phosphatase inhibitor cocktail II (Sigma). After centrifugation, samples of supernatant were mixed with NuPage™ sample reducing agent, heated to 70°C for 10 min then separated by SDS-PAGE using a 10% Bis-Tris gel and NuPage™ gel system (Invitrogen). Proteins were transferred onto nitrocellulose membranes using NuPage™ transfer buffer (Invitrogen) and probed with the 1A11 antibody as previously described [47].

### Statistical analysis

For statistical analysis, Chi-square tests and student t-tests were performed using Microsoft Excel Software. A p-value of <0.05 was considered statistically significant. Dr David Cairns (Leeds University) gave invaluable statistical advice.

## Supporting Information

Figure S1 **Specificity of EB1 antibody 1A11 demonstrated in a full length Western blot.** In addition to the full length Western profile of the original blot (A) another Western was carried out to confirm binding patterns (B). NRK-52E cells were lysed and prepared in sample buffer as previously described. Lysates were run on a 4–12% SDS-PAGE gel (Invitrogen) and proteins were transferred to a PVDF membrane. The membrane was blocked with 5% skimmed milk powder (Marvel) in PBS for 2 h on a rocker and incubated with the EB1 antibody 1A11 (1/100) in 1% Marvel-PBS overnight at 4°C. The membrane was washed 5 times for 5 min with PBS and then incubated with goat anti-mouse antibody (1/10000) (Pierce) for 1 h at 21°C. After 5 further washes Pierce Femto Kit reagents were added to the membrane and allowed to react for 5 min. Arrow indicates the endogenous EB1 protein in the whole lysate. Endogenous EB1 is present in NRK52-E cells as is recognized as an approx 30 kDa protein in the full length Western blot by the EB1 antibody. We also noted one additional band at around 55 kDa in the blot. 1A11 has been commercially available for some time and Western blot images in for example the product sheet supplied by Cell Signaling Technology (EB-1 1A11/4, cat. number 2164) appear to reveal some additional non specific bands). Furthermore, an accessory band at around 55 kDa has been shown in at least two other EB1 antibodies, for example abcam MAPRE1 antibody, ab50188, and Novus Biological antibody NBP1 28753.
(TIF)

Figure S2 **Diagram of the EB1 protein indicating putative domains and binding sites for EB1 interactants including APC (red), p150Glued (blue), MCAK (orange) and CLIP-170 (purple).** The antibody symbol indicates the location of the region for the epitope recognised by 1A11. The CLIP-170 binding region encompasses aa 125–168. The C-terminal tyrosine is essential. MCAK binds within the last 84 aa of EB1. The last 27 aa are essential.
(TIF)

Figure S3 **Microinjection of two antibodies, 1A11 and ALI 12–28, does not lead to an additive negative effect on mitotic NRK-52E cells.** Mitotic NRK-52E cells (n = 17) were microinjected as described under Materials and Methods with an antibody mixture containing EB1 antibody 1A11 and APC antibody ALI 12–28 at a needle concentration of 1 mg/ml each. Microinjected cells were imaged and analysed as before. A. The majority of microinjected cells complete mitosis. B. Microinjection of a combination of both antibodies leads to a delay in cytokinesis but not at other time points during mitosis. C. Early mitotic cortical blebbing is reduced after co-injection of 1A11 and ALI 12–28. D. Late mitotic cortical blebbing remains at high levels after co-injection of 1A11 and ALI 12–28. E. The majority of the cells were correctly aligned at TP1 and had all aligned correctly by TP 2. F. Severe uneven spindle pole movement was observed in 25% of the cells microinjected with a combination of 1A11 and ALI 12–28 (1- 1A11; 2- ALI-12-28; 3- 1A11 and 12–28 combined).
(TIF)

Table S1 **Summary of blebbing in mitotic NRK-52E cells microinjected with 1A11, ALI 12–28, C-APC 9.9 and C-APC 28.9.** Anaphase specific blebbing was only observed in cells microinjected with 1A11 whereas blebbing was observed from prophase or prometaphase onwards in cells microinjected with the APC antibodies.
(DOC)

Movie S1 **Video of a mitotic NRK-52E cell microinjected during prophase with the control antibody 4 U.** Note the tight chromosomal congression at the metaphase plate, the absence of cortical blebbing and the smooth, symmetrical chromosomal separation during anaphase. Images were obtained using 1×1 binning and exposure times of less than 250 ms/frame with a time-lapse interval of 30 s. For presentation as movies, image series were saved as uncompressed AVI files then cropped, compressed and converted into QuickTime movies using Adobe ImageReady 7. Stills from this movie are presented in Figure 3A.
(MOV)

Movie S2 **Video of mitotic NRK-52E cell microinjected with the EB1-specific antibody 1A11.** Note the comparative looseness of chromosomal congression before anaphase onset, the asymmetric movement of the separating chromosomes and the cortical blebbing during anaphase. Images were obtained using 1×1 binning and exposure times of less than 250 ms/frame with a time-lapse interval of 30 s, for up to 2 h. For presentation as movies, image series were saved as uncompressed AVI files then cropped, compressed and converted into QuickTime movies using Adobe ImageReady 7. Stills from this movie are presented in Figure 3B.
(MOV)

Movie S3 **Video of mitotic NRK-52E cell microinjected with the APC-specific antibody ALI 12–28.** Note the comparative looseness of chromosomal congression before anaphase onset. Images were obtained using 1×1 binning and exposure times of less than 250 ms/frame with a time-lapse interval of 30 s, for up to 2 h. For presentation as movies, image series were saved as uncompressed AVI files then cropped, compressed and converted into QuickTime movies using Adobe ImageReady 7. Stills of this movie are presented in Figure 3C.
(MOV)

Movie S4 **Video of mitotic NRK-52E cell microinjected with the APC-specific antibody C-APC 9.9.** Note the cortical blebbing initiated during metaphase and the comparative looseness of chromosomal congression before anaphase onset. Images were obtained using 1×1 binning and exposure times of less than 250 ms/frame with a time-lapse interval of 30 s, for up to 2 h. For presentation as movies, image series were saved as uncompressed AVI files then cropped, compressed and converted into QuickTime movies using Adobe ImageReady 7. Stills of this movie are presented in Figure 3D.
(MOV)

Movie S5 **Video of mitotic NRK-52E cell microinjected with the APC-specific antibody C-APC 28.9.** Note the cortical blebbing initiated during metaphase and the comparative looseness of chromosomal congression before anaphase onset. Images were obtained using 1×1 binning and exposure times of less than 250 ms/frame with a time-lapse interval of 30 s, for up to 2 h. For presentation as movies, image series were saved as uncompressed AVI files then cropped, compressed and converted into QuickTime movies using Adobe ImageReady 7. Stills of this movie are presented in Figure 3E.
(MOV)

Movie S6 **Video of mitotic NRK-52E cell microinjected with the EB1-specific antibody 1A11.** Note spindle misalignment and misplacement at metaphase that is corrected by spindle movements during anaphase. Images were obtained using 1×1 binning and exposure times of less than 250 ms/frame with a time-lapse interval of 30 s, for up to 2 h. For presentation as movies, image series were saved as uncompressed AVI files then cropped, compressed and converted into Quicktime movies using Adobe ImageRead 7. Stills of this movie are presented in Figure 5C.
(MOV)

Movie S7 **Video of a mitotic NRK-52E cell microinjected during prophase with the control GST protein only.** Note the tight chromosomal congression at the metaphase plate, the absence of cortical blebbing and the smooth, symmetrical chromosomal separation during anaphase. Images were obtained using 1×1 binning and exposure times of less than 250 ms/frame with a time-lapse interval of 30 s. For presentation as movies, image series were saved as uncompressed AVI files then cropped, compressed and converted into QuickTime movies using Adobe ImageReady 7. Stills from this movie are presented in Figure 12.
(MOV)

Movie S8 **Video of a mitotic NRK-52E cell microinjected during prophase with the EB1-C84 GST fusion protein.** Note the diffuse chromosomal congression at the metaphase plate, cortical blebbing and the asymmetrical chromosomal separation during anaphase and onwards. Also note the formation of an ectopic cleavage furrow. Images were obtained using 1×1 binning and exposure times of less than 250 ms/frame with a time-lapse interval of 30 s. For presentation as movies, image series were saved as uncompressed AVI files then cropped, compressed and converted into QuickTime movies using Adobe ImageReady 7. Stills from this movie are presented in Figure 12.
(MOV)
