# Supplementary figures and images for: EB1 Is Required for Spindle Symmetry in Mammalian Mitosis
Source: PLoS One. 2011 Dec 21;6(12):e28884. doi: 10.1371/journal.pone.0028884 (PMC3244432; doi:10.1371/journal.pone.0028884)

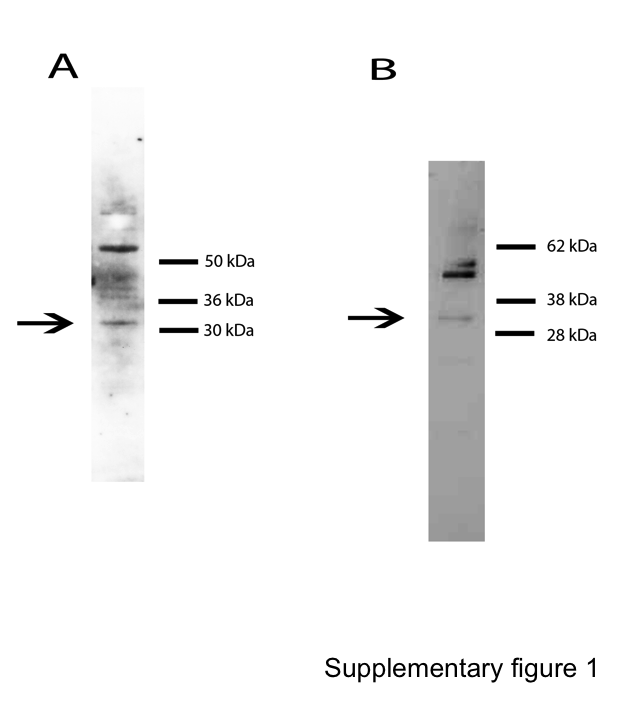

Supplement: Figure S1 — Specificity of EB1 antibody 1A11 demonstrated in a full length Western blot. In addition to the full length Western profile of the original blot (A) another Western was carried out to confirm binding patterns (B). NRK-52E cells were lysed and prepared in sample buffer as previously described. Lysates were run on a 4–12% SDS-PAGE gel (Invitrogen) and proteins were transferred to a PVDF membrane. The membrane was blocked with 5% skimmed milk powder (Marvel) in PBS for 2 h on a rocker and incubated with the EB1 antibody 1A11 (1/100) in 1% Marvel-PBS overnight at 4°C. The membrane was washed 5 times for 5 min with PBS and then incubated with goat anti-mouse antibody (1/10000) (Pierce) for 1 h at 21°C. After 5 further washes Pierce Femto Kit reagents were added to the membrane and allowed to react for 5 min. Arrow indicates the endogenous EB1 protein in the whole lysate. Endogenous EB1 is present in NRK52-E cells as is recognized as an approx 30 kDa protein in the full length Western blot by the EB1 antibody. We also noted one additional band at around 55 kDa in the blot. 1A11 has been commercially available for some time and Western blot images in for example the product sheet supplied by Cell Signaling Technology (EB-1 1A11/4, cat. number 2164) appear to reveal some additional non specific bands). Furthermore, an accessory band at around 55 kDa has been shown in at least two other EB1 antibodies, for example abcam MAPRE1 antibody, ab50188, and Novus Biological antibody NBP1 28753. (TIF) [file pone.0028884.s001.tif]

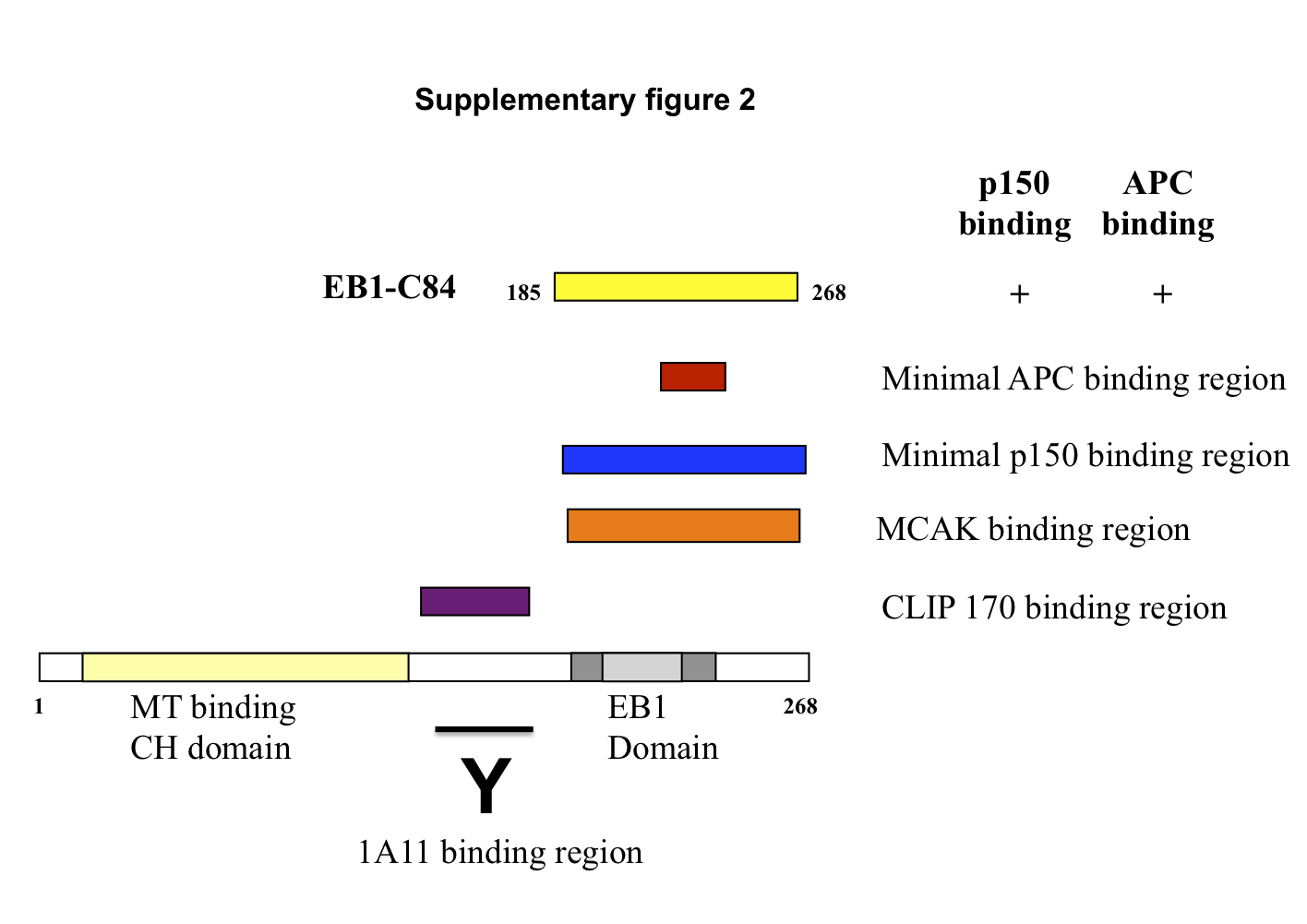

Supplement: Figure S2 — Diagram of the EB1 protein indicating putative domains and binding sites for EB1 interactants including APC (red), p150Glued (blue), MCAK (orange) and CLIP-170 (purple). The antibody symbol indicates the location of the region for the epitope recognised by 1A11. The CLIP-170 binding region encompasses aa 125–168. The C-terminal tyrosine is essential. MCAK binds within the last 84 aa of EB1. The last 27 aa are essential. (TIF) [file pone.0028884.s002.tif]

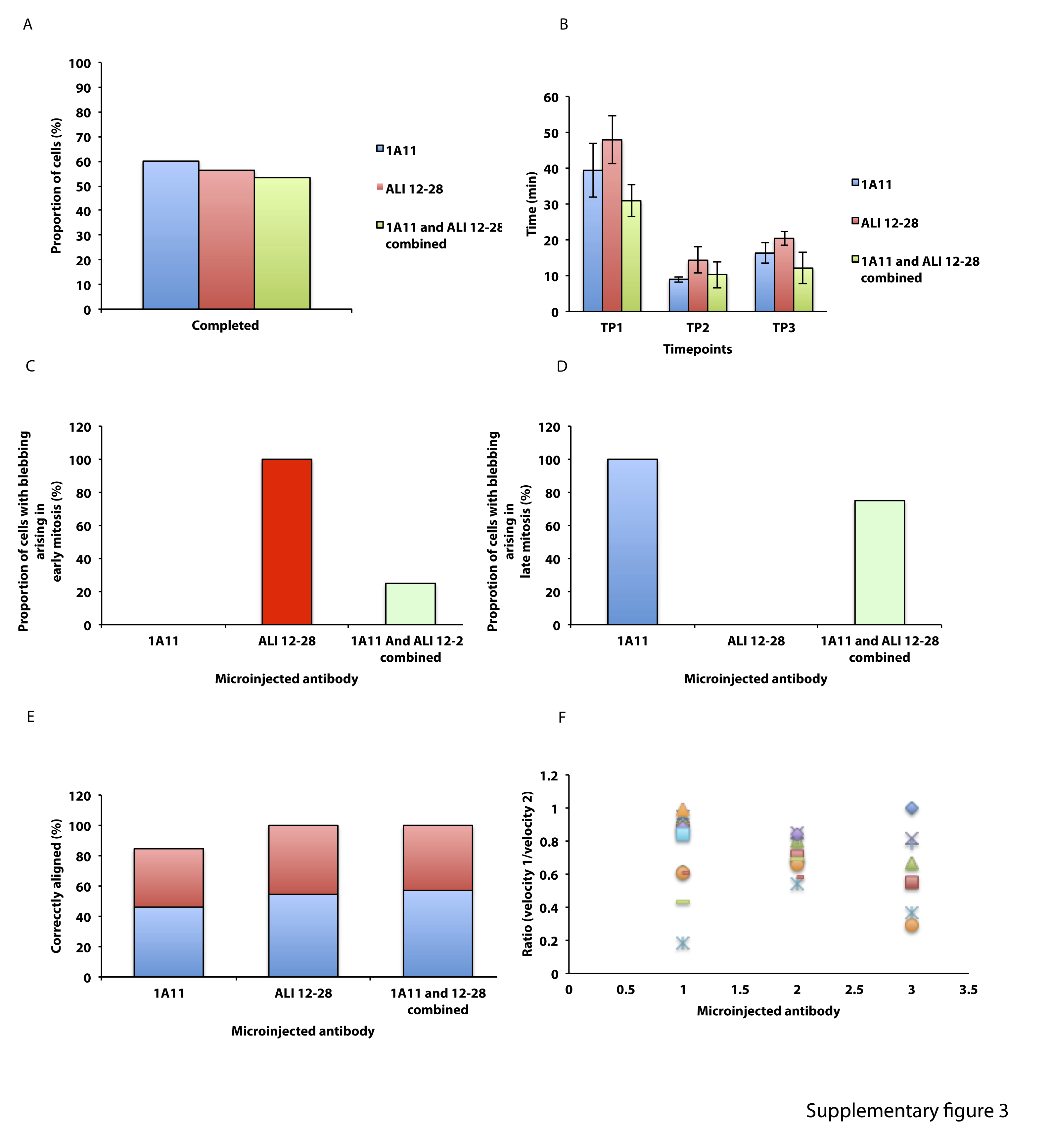

Supplement: Figure S3 — Microinjection of two antibodies, 1A11 and ALI 12–28, does not lead to an additive negative effect on mitotic NRK-52E cells. Mitotic NRK-52E cells (n = 17) were microinjected as described under Materials and Methods with an antibody mixture containing EB1 antibody 1A11 and APC antibody ALI 12–28 at a needle concentration of 1 mg/ml each. Microinjected cells were imaged and analysed as before. A. The majority of microinjected cells complete mitosis. B. Microinjection of a combination of both antibodies leads to a delay in cytokinesis but not at other time points during mitosis. C. Early mitotic cortical blebbing is reduced after co-injection of 1A11 and ALI 12–28. D. Late mitotic cortical blebbing remains at high levels after co-injection of 1A11 and ALI 12–28. E. The majority of the cells were correctly aligned at TP1 and had all aligned correctly by TP 2. F. Severe uneven spindle pole movement was observed in 25% of the cells microinjected with a combination of 1A11 and ALI 12–28 (1- 1A11; 2- ALI-12-28; 3- 1A11 and 12–28 combined). (TIF) [file pone.0028884.s003.tif]
