# Supplementary material for: EB1 Is Required for Spindle Symmetry in Mammalian Mitosis
Source: PLoS One. 2011 Dec 21;6(12):e28884. doi: 10.1371/journal.pone.0028884 (PMC3244432; doi:10.1371/journal.pone.0028884)
Supplement: Table S1 — Summary of blebbing in mitotic NRK-52E cells microinjected with 1A11, ALI 12–28, C-APC 9.9 and C-APC 28.9. Anaphase specific blebbing was only observed in cells microinjected with 1A11 whereas blebbing was observed from prophase or prometaphase onwards in cells microinjected with the APC antibodies. (DOC) [file pone.0028884.s004.doc]

| **Microinjected antibody** | **Mitotic stage when blebbing first observed** |
| --- | --- |
| 4U | No blebbing |
| 1A11 | Anaphase |
| ALI 12-28 | Prophase |
| CAPC 9.9 | Prophase/prometaphase |
| CAPC 28.9 | Prophase/prometaphase |

Supplementary table 1
